# Synovial inflammatory macrophage-derived extracellular vesicles exacerbate cartilage lesions with a FMRP-selectively sorted manner in osteoarthritis

**DOI:** 10.1038/s41413-025-00502-4

**Published:** 2026-02-17

**Authors:** Shu Zhao, Jian Wang, Mengxin Xue, Baitong Wu, Lanyue Sheng, Yi Wen, Guangming Wang, Jianxing Song, Dajiang Du, Jun Xu

**Affiliations:** 1 Shanghai Key Laboratory of Anesthesiology and Brain Functional Modulation, Clinical Research Center for Anesthesiology and Perioperative Medicine, Translational Research Institute of Brain and Brain-Like Intelligence, Department of Anesthesiology and Perioperative medicine, Shanghai Fourth People’s Hospital, School of Medicine, Tongji University, Shanghai, PR China; 2 East Hospital, Stem Cell Research Center, School of Medicine, Tongji University, Shanghai, PR China; 3 Department of Orthopedic Surgery, Shanghai Sixth People’s Hospital Affiliated to Shanghai Jiao Tong University School of Medicine, Shanghai, PR China

**Keywords:** Pathogenesis, Bone

## Abstract

Osteoarthritis (OA) is an aging-related degenerative joint disease without effective therapies. In the early stage of OA, mild synovitis has been reported to induce cartilage lesions. A better understanding of crosstalk between synovial macrophages and chondrocytes are being developed to discover new OA therapeutics. Here, we identified that the extracellular vesicles (EVs) derived from synovial pro-inflammatory macrophages regulated the autophagy function of chondrocytes, induced the onset of cartilage degeneration in normal joints. Mechanistically, the active transfer of miR-155-5p via EVs from synovial pro-inflammatory macrophages to chondrocytes accelerates cartilage degeneration by suppressing GSK-3β/mTORC1 axis-mediated autophagy function during OA progression. Deleting miR-155 from synovial pro-inflammatory macrophages relieved cartilage lesions and synovitis in OA mice. On the other hand, Fragile X mental retardation protein (FMRP) selectively sorted miR-155-5p into EVs derived from synovial pro-inflammatory macrophages, and the levels of plasma EVs FMRP were closely related to OA progression, suggesting the potential candidate for diagnostic OA biomarkers. Based on these findings, we developed engineering EVs with MAP (pro-inflammatory macrophages-affinity peptide) derived from adipose-derived stromal cells (ADSCs) as the antagomiR-155-5p delivery vehicles which exhibited superior therapeutic effects on synovitis and injured cartilage in the surgery-induced OA rats. Furthermore, MAP-ADSCs-EVs were proved to target the polarization of synovial pro-inflammatory macrophages in the clinical OA samples. Collectively, our study indicates that plasma EVs FMRP and engineered MAP-ADSCs-EVs targeting synovial pro-inflammatory macrophages represent potential novel therapeutic strategy for the progression of OA.

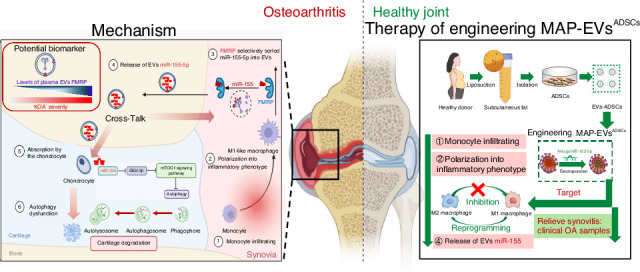

## Introduction

Osteoarthritis (OA) is the most prevalent aging-related degenerative joint disorder, causing pain and functional disability, affecting millions of individuals globally.^1,2^ However, clinical therapies for OA treatments only relieve pains and symptoms, and disease-altering therapies to prevent or postpone OA progression successfully are still limited.^3,4^ The primary reason is the complex and limited understanding of the mechanism.^1^ Therefore, it is critical to study pathogenesis further and develop novel therapeutic agents to prevent the onset and development of OA.

OA is a progressive degenerative disorder that affects the entire joint tissues and is mainly characterized by articular cartilage erosion, subchondral bone sclerosis, osteophyte formation, and synovial inflammation (synovitis).^5^ Synovitis is a predictive factor of the structural progression of knee OA.^6–8^ Low-grade synovitis has been reported to induce the onset and progression of chondropathy and precede cartilage degradation, even though the joint injury cannot be detected clinically.^9–11^ The significant increase of immune cells in synovitis, including macrophage, B cell, B cell memory, B cell naïve, B cell plasma and mast cell, have been reported to be related to the occurrence and development of inflammation in OA progression.^12^ The infiltrating macrophages, including pro-inflammatory and anti-inflammatory macrophages, have been reported to participate in the initiation and development of synovitis.^13^ The OA microenvironment stimuli, including pathogen-associated molecular patterns, danger-associated molecular patterns and inflammasome, recruited quantities of monocytes that migrated from the bone marrow into the synovium and subsequently polarized to the pro-inflammatory phenotype, which would impair joint homeostasis.^14,15^ In addition, a series of abnormal molecules from synovial pro-inflammatory macrophages, including pro-inflamatory cytokines like interleukin-6 (IL-6), interleukin-1β (IL-1β), tumor necrosis factor-ɑ (TNF-ɑ), the elevated R-spondin-2,^16^ phosphoglycerate mutase 5 (PGAM5),^17^ Src-homology2-containing protein tyrosine phosphatase 2 (SHP2),^18^ have been identified to regulate the synovium-cartilage cross-talk to aggregate cartilage degeneration in OA progression. The mechanism by which pro-inflammatory macrophage disrupt cartilage homeostasis is fundamental for understanding the pathogenesis mediated by synovium-cartilage cross-talk during the onset progression of OA and require futher in-depth investigation.

Extracellular vesicles (EVs) rang in diameter from 40 to 150 nm and are released by all kinds of cells. EVs act as a unique intercellular shuttle, transferring proteins, lipids, and nucleic acids (mostly microRNAs) between cells.^19^ EVs have been engaged in several physiological and pathological mechanisms. Numerous studies have demonstrated that EVs in synovial fluid play crucial roles in the pathogenesis of joint disorders, including rheumatoid arthritis and OA.^20–22^ Up to now, the two researches have explored the EVs released from RAW264.7 elevated cartilage catabolism in the collagenase-induced OA mice model.^23,24^ However, the contents in the EVs released from pro-inflammatory macrophages exacerbate cartilage lesion in OA progression, has been seldomly reported. Thus, the effects of the EVs from synovial pro-inflammatory macrophages on normal cartilage homeostasis and the contents of the EVs induced OA disease were investigated in this study.

The EVs biomolecules would be changed upon the cellular inflammation, and specific processes exist to separate molecules that are intended for EVs export.^25^ However, the mechanism by which miRNAs are sorted into EVs released from synovial pro-inflammatory macrophage remains largely unknown. The sorting of EVs miRNA has been described to be impacted by lipids/ceramide, direct miRNA alteration, and elements of the multivesicular bodies biogenesis cascade.^26,27^ RNA binding proteins (RBPs) are important participants in EVs miRNA sorting.^28^ The mechanisms by which RBPs mediate miRNA sorting into EVs released from synovial pro-inflammatory macrophages remain elusive.

The objective of this research was to examine the influences of EVs produced by synovial pro-inflammatory macrophages on normal chondrocyte catabolism, the unique contents in the EVs, and the molecular mechanism underlying their pathological function in the progression of OA. Specifically, we investigated the profile of miRNAs in circulating synovial pro-inflammatory macrophages-originated EVs in OA models, then looked into how the active transport of synovial pro-inflammatory macrophages derived EVs miRNAs led to the OA development, and evaluated the therapeutic potential of engineering EVs derived from ADSCs targeting synovial pro-inflammatory macrophages as miRNA delivery vehicles on OA models.

## Results

### Synovial pro-inflammatory macrophages aggravate cartilage damage in the progression of OA via an EVs-mediated manner

First of all, medial meniscus destabilization (DMM) and anterior cruciate ligament transection (ACLT) operation of the knee was carried out in 8-week-old male SD rats to develop the surgery-induced OA model (Fig. S1A). We investigated synovial inflammation and macrophage phenotypes in the OA rat model to study synovial changes during OA development. In the synovial tissue of OA rats, we observed significant synovial hyperplasia and extensive cell infiltration, along with markedly higher synovitis scores compared to control knees (Fig. S1B, C), consistent with previous research findings.^29,30^ Next, we characterized the morphology and abundance of macrophages in the synovial region of OA rats. Compared to controls, there were significant increases in F4/80-positive cells (a macrophage marker) and decreases in CD206-positive cells (an M2-like macrophage marker) in the intimal lining layer of OA synovial tissue (Fig. S1D, F). In OA synovium, the proportion of cells positive for the M1-like macrophage marker iNOS increased significantly (Fig. S1E, G). These results indicate that macrophages exhibit increased M1-like polarization and decreased M2-like polarization in OA synovial tissue, suggesting their potential role in cartilage lesions of OA progression.

**Fig. 1:**
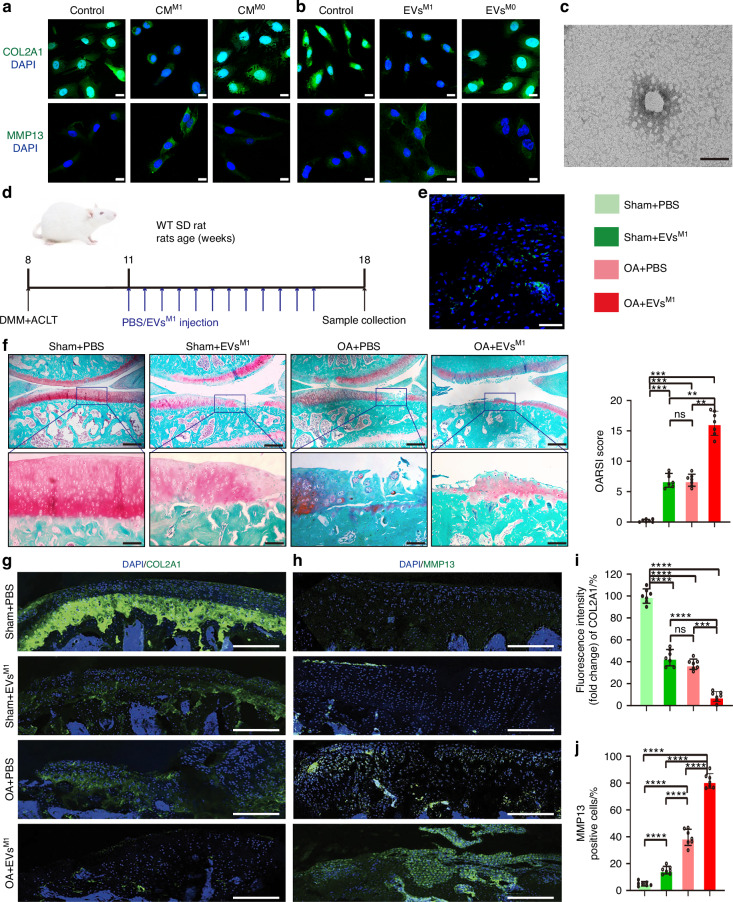
Synovial pro-inflammatory macrophages-derived EVs aggravate cartilage damage in OA progression. **a** Representative images of COL2A1 and MMP13 were assessed by IF staining in C28/I2 cells treated with conditioned medium from THP-1 cells. Scale bar: 20 μm. DAPI: 4,6-diamidino-2-phenylindole. **b** Representative images of COL2A1 and MMP13 were assessed by IF staining in C28/I2 cells treated with EVs from THP-1 cells. Scale bar: 20 μm. DAPI: 4,6-diamidino-2-phenylindole. **c** Representative transmission electronic microscope (TEM) images of EVs derived from M1-polarized BMDMs in rat models. Scale bar: 100 nm. **d** A schematic diagram illustrating the experimental design. **e** The representative confocal images of DiO-labeled EVs derived from M1-polarized BMDMs in tibial cartilage in OA rats at 1 week after surgery. Scale bar: 100 μm. **f** SO & FG staining in keen joints of sham or OA rats treated with PBS or EVs^M1^ (up), scale bar: 500 μm. Higher magnification images show dramatic articular cartilage changes (down), scale bar: 100 μm. (left) Quantification of OARSI score was performed using histological sections. *n* = 7 for each group. (right) IF staining of knee joint sections showing expression of COL2A1 (**g**) and MMP13 (**h**) in articular cartilage. Scale bar: 100 μm. DAPI: 4,6-diamidino-2-phenylindole. Quantification of COL2A1 (**i**) and MMP13 (**j**) expression in articular cartilage with IF staining. *n* = 7 for each group. One-way ANOVA &Tukey HSD post hoc test (normal distribution) and Kruskal-Wallis Test & Dunn’s test (non-normal distribution) were used for multiple comparisons. * *P* < 0.05, ** *P* < 0.01, ***P < 0.001, **** *P* < 0.000 1, ns not significant

Second, the significance of synovial pro-inflammatory macrophages in OA microenvironment and their possible impacts on chondrocytes were investigated in vitro. The THP-1 cells were induced to M0-like macrophages using PMA, then induced to M1-like macrophages using LPS + ATP (Fig. S2A) (full inflammasome activation, a primary activation mechanism of synovial macrophages), according to the methods.^31^ The effect of cell culture supernatants from M1/M0-like THP-1 cells on C28/I2 was investigated in the co-culture trials. The IF results showed that the conditioned medium of M1-like macrophages (CM^M1^) significantly decreased the expression of cartilage type II collagen A1 (COL2A1), and increased the expression of matrix metallopeptidase 13 (MMP13) in C28/I2 cells, compared to the conditioned medium of M0-like macrophages (CM^M0^) (Fig. 1a, S1H, S1I). Considering EVs as the important paracrine elements to mediate cell-to-cell communication, then EVs derived from M0-like-THP-1 (EVs^M0^) and M1- like-THP-1 (EVs^M1^) conditioned medium were extracted, identified (Fig. S2B, C) and then treated with C28/I2 cells. EVs^M1^ markedly decreased the expression of COL2A1 and increased the expression of MMP13 in C28/I2 cells (Fig. 1b, S1J, S1K).

Furthermore, the significance of synovial pro-inflammatory macrophages in the OA microenvironment and their possible impacts on chondrocytes were investigated in vivo. Recent studies have demonstrated that macrophages infiltrated into the synovium are mainly migratory bone marrow-derived macrophages (BMDMs), which are recruited and differentiate into anti-inflammatory M2-like or pro-inflammatory M1-like phenotypes after homing to inflammatory tissues with OA progression.^13,32^ Then, the effects of EVs from pro-inflammatory BMDMs with OA progression were investigated in vivo. First of all, the BM-derived monocytes from OA rats femurs were separated, and the monocytes carried the markers of CD45^+^ and CD11b^+^ were sorted using flow cytometry (Fig. S2D). Then, the sorted BM-derived monocytes were induced to M1-like polarization (Fig. S2E). Differential ultrafiltration was used to isolate EVs derived from M1-like BMDMs (EVs^M1-BMDMs^), and the typical characteristics of EVs were identified using various experiments (Fig. 1c; Fig. S2F). DMM and ACLT operation of the knee was carried out in 8-week-old male SD rats to develop the surgery-induced OA model. Initially, DiO-labeled EVs^M1-BMDMs^ were injected into the surgical joint of rats to evaluate their ability to penetrate the injured articular cartilage (Fig. 1d). Following the intra-articular injection of DiO-labeled EVs, areas of damaged cartilage exhibited distinct green fluorescent patches under a fluorescent microscope (Fig. 1e). Second, following the protocol outlined in Fig. 1d, EVs^M1-BMDMs^ diluted in PBS (EVs^M1^) or PBS as negative control were administered to sham or OA rats. Safranin O and fast green (SO & FG) staining showed that the articular cartilage exhibited evident degradation following OA surgery, as depicted in the representative image in Fig. 1f. Compared to the PBS group, the EVs^M1^ group showed more pronounced cartilage breakdown and proteoglycan loss with reduced cartilage surface, demonstrated with the results of Osteoarthritis Research Society International (OARSI) scores (Fig. 1f). IF analysis revealed that EVs^M1^ injection markedly reduced COL2A1 expression (Fig. 1h, i) and increased MMP13 expression in the cartilage of OA rats, in contrast to the PBS group (Fig. 1h, j).

Taken together, we concluded that EVs derived from pro-inflammatory macrophages, which could mediate the interaction between synovitis and cartilage lesions, may be responsible for the decline in chondrocyte homeostasis of normal cartilage in the progression of OA.

### Synovial pro-inflammatory macrophages-derived EVs downregulate the autophagy function of chondrocytes in the progression of OA

To determine the exact mechanism via which synovial pro-inflammatory macrophages-derived EVs promote ECM degradation and reduce cartilage function, the gene expression in articular cartilage was examined. RNA-seq analysis was used to examine the gene expression of cartilage specimens in both the EVs^M1^-treated group and PBS-treated group (control) in OA rats of the former trial (Fig. 1d). A Heatmap of OA rats treated with EVs^M1^ revealed a variety of identified genes that were down-regulated genes (3521) and up-regulated genes (1990) (*P* < 0.05) (Fig. S3A). KEGG enrichment analysis was performed based on the genes that showed differential expression. The information revealed that several critical biological processes connected to autophagy, such as endocytosis and phagosome process, were exquisitely controlled within the cell (Fig. S3B). In addition, a thorough analysis of the RNA-seq data was conducted using gene set enrichment analysis. It is interesting to note that the GSEA results, which were strongly connected with our findings that EVs^M1^ exacerbated the cartilage deterioration of OA rats, showed that EVs^M1^ treatment continuously negatively impacted the cartilage development, extracellular matrix process and chondrocyte differentiation (Fig. S2G). We looked into whether the impact of EVs^M1^ on cartilage was connected to the autophagy process to validate the RNA-seq data analysis results previously reported. The TEM test, the gold standard for assessing autophagy, was used to identify the autolysosomes and autophagosomes in the cartilage of joints. The findings demonstrated that the autophagic vesicle in the cartilage of OA rats treated with EVs^M1^ was considerably reduced in comparison to the PBS group (Fig. S3C, D), suggesting that the OA group that received the EVs^M1^ injection had suppressed autophagy levels.

Furthermore, the effect of EVs derived from synovial pro-inflammatory macrophages on chondrocyte autophagy was detected in vitro. Autophagic flux was measured in C28/I2 cell lines transfected with a tandem fluorescent mRFP-GFP-LC3 adenovirus. RFP signals only (red dots) show the existence of autolysosomes, while the colocalization of GFP and RFP signals (yellow dots) demonstrates the lack of autophagosome-lysosome or phagophore fusion. CM^M1^ considerably reduced the autophagosomes and autolysosomes formation in C28/I2 cells, compared to CM^M0^ (Fig. S3E, F). Meanwhile, EVs^M1^ substantially inhibited the autophagosomes and autolysosomes formation in C28/I2 cells, compared to EVs^M0^ (Fig. S3G, H). The above results indicated that EVs derived from synovial pro-inflammatory macrophages aggravate ECM degradation and hinder cartilage function recovery by suppressing chondrocyte autophagy in OA progression.

### EVs miR-155-5p derived from synovial pro-inflammatory macrophages regulate the autophagy function of chondrocytes

**Fig. 2:**
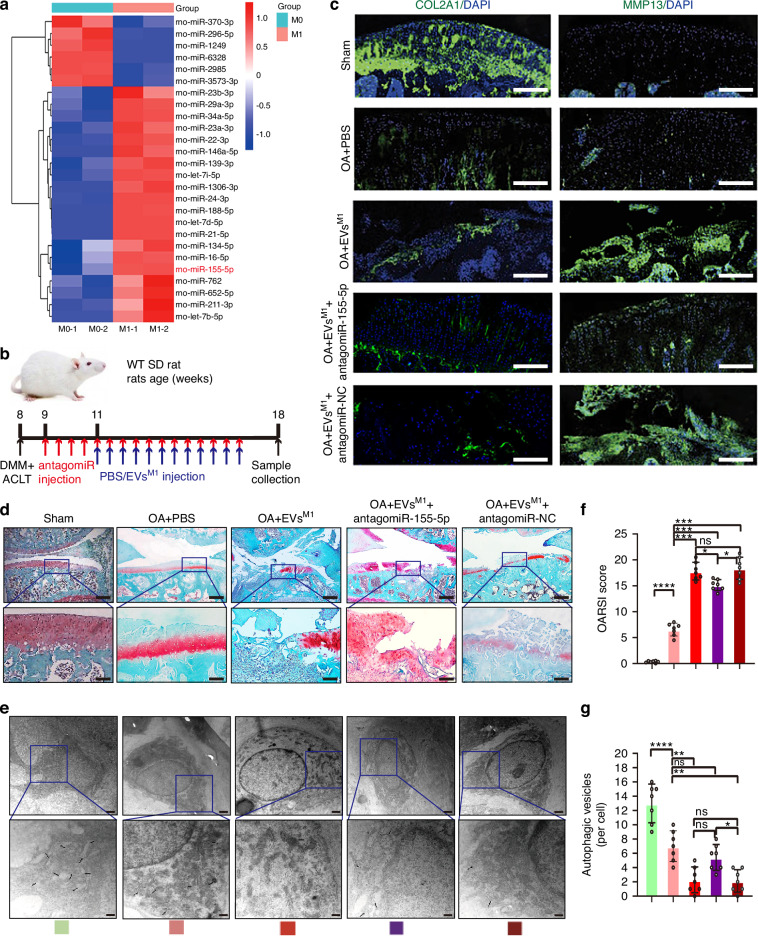
Synovial pro-inflammatory macrophages-derived EVs miR-155-5p regulates autophagy function of chondrocytes. **a** Heatmap showing the hierarchical cluster of differential miRNA enrichments in EVs derived from M0 and M1-polarized BMDMs sorted from CD45^+^, CD11b^+^ monocytes between sham and OA rats models. *n* = 2 for each group. **b** A schematic diagram illustrating the experimental design. **c** IF staining of knee joint sections showing expression of COL2A1 and MMP13 in articular cartilage. Scale bar: 100 μm. DAPI: 4,6-diamidino-2-phenylindole. **d** SO & FG staining in keen joints of sham or OA rats treated with PBS, EVs^M1^, antagomiR-155-5p, and antagomiR-NC (up), scale bar: 500 μm. Higher magnification images show dramatic articular cartilage changes (down), scale bar: 100 μm. **e** Transmission electron microscopy (TEM) image of autophagic vesicles in articular cartilage of OA rats. The black arrow indicates the cell bilayer membrane structure of autophagic vesicles. Scale bar: 1 μm. **f** Quantification of OARSI score was performed using histological sections. *n* = 7 for each group. **g** Quantification of autophagic vesicles in articular cartilage of OA rats with TEM detection. *n* = 7 for each group. One-way ANOVA &Tukey HSD post hoc test (normal distribution) and Kruskal-Wallis Test & Dunn’s test (non-normal distribution) were used for multiple comparisons. * *P* < 0.05, ** *P* < 0.01, *** *P* < 0.001, **** *P* < 0.000 1, ns not significant

To explore the underlying mechanism by which synovial pro-inflammatory macrophages aggravate cartilage damage in OA progression, the miRNA enrichment in EVs derived from synovial macrophages between sham and OA rats was examined. As BM-derived macrophages contributed to the pool of synovial macrophages during OA progression, the CD45^+^CD11b^+^ monocytes from BM cells between sham and OA rats were sorted according to the strategy shown in Fig. S2D. Then the sorted monocytes were induced to M0-like (presented synovial macrophages in the sham model) and M1-like polarization (presented synovial pro-inflammatory macrophages in the OA model), separately. Using the miRNA quantitative PCR array analysis, a series of miRNAs, including let-7b-5p, miR-211-3p, miR-652-5p, miR-762, miR-155-5p, miR-16-5p, were significantly upregulated in EVs derived from M1-like BMDMs of OA rats as compared to EVs derived from M0-like BMDMs of sham rats, which we defined as EVs^M1-BMDMs^-miRNAs (Fig. 2a). Then bioinformatics analysis of the our results (miRNAs of EVs^M1-BMDMs^) with GSE33453 (miRNAs of M0/M1-like BMDMs) and GSE175961 (miRNAs of the cartilage between KOA patients and healthy controls) showed that only miR-155-5p is significantly increased in EVs^M1-BMDMs^, M1-like macrophages and KOA patients compared with EVs^M0-BMDMs^, M0-like macrophages and healthy controls (Fig. S4A). Consistently, miR-155-5p levels is markly higher in EVs^M1-BMDMs^ than EVs^M0-BMDMs^, while decreased in M1-like BMDMs than in M0-like BMDMs (Fig. S4B), suggesting that the EVs miR-155-5p secretion was so efficient that intracellular miR-155-5p levels of BMDMs declined. As EVs could transfer quantities of miRNAs to recipient cells and play an vital role in the function of recipient cells,^33^ we supposed that the miR-155-5p in EVs^M1-BMDMs^ may be transferred to chondrocytes to impact chondrocyte function. The co-culture trials showed that miR-155-5p levels were significantly enhanced in EVs^M1-BMDMs^-treated rat chondrocytes, demonstrating that miR-155 could be delivered from synovial pro-inflammatory macrophages to chondrocytes through EVs (Fig. S4B, S4F). Notably, in vivo trial showed the miR-155-5p levels of cartilage and synovium were increased in the OA rat group with OA progression, compared to the sham group; the miR-155-5p levels of synovium were even markedly higher than that of cartilage in OA rat group (Fig. S4C). Interestingly, the results of clinical samples showed that the miR-155-5p level was also upregulated in both synovium and cartilage from OA patients with total hip joint replacement surgeries as compared to the control human samples; the miR-155-5p level of synovium was even significantly enhanced than that of cartilage in OA patients (Fig. S4G).

To determine whether EVs miR-155-5p derived from synovial pro-inflammatory macrophages participated in cartilage destruction, we used antagomiR-155-5p in the OA rat model. EVs^M1^ with or without antagomiR-155-5p were intra-articularly injected into the surgical-operated OA rats respectively (Fig. 2b). After 10 weeks of OA surgery, knee joints were sampled for histological analysis. AntagomiR-155-5p inhibited the effect of EVs^M1^-mediated cartilage destruction in OA rats (Fig. 2d, f), in line with the results of IF which showed that EVs^M1^ could increase the expression of MMP13 and decrease the expression of COL2A1, which were reversed by intra-articular injection of antagomiR-155-5p (Fig. 2c, S4D, S4E). To conclude, the aggravation of cartilage destruction induced by EVs^M1^ highly depended on miR-155-5p. Then, to clarify the mechanism of the effect of EVs^M1^ on cartilage, we detected the autophagy process of cartilage mediated by EVs^M1^ and antagomiR-155-5p in OA rats. The TEM results showed that the autophagic vesicles in the cartilage of OA rats treated with EVs^M1^ and antagomiR-155-5p were significantly increased compared to the antagomiR-NC group (Fig. 2e, g), indicating the elevated autophagy levels treated with antagomiR-155-5p. Moreover, the levels of related autophagic proteins, including beclin1, LC3A/B, Atg3, Atg7, and Lamp1, were significantly enhanced in the articular cartilage treated with EVs^M1^ and antagomiR-155-5p, compared to EVs^M1^ and antagomiR-NC (Fig. S4H).

The above results indicated that EVs derived from synovial pro-inflammatory macrophages could reduce chondrocyte autophagy, aggravate cartilage ECM degradation, and hinder cartilage function recovery in OA progression. This was highly dependent on EVs miR-155-5p.

### Synovial pro-inflammatory macrophages-derived EVs miR-155-5p targets GSK-3β/mTORC1 axis in OA chondrocytes

**Fig. 3:**
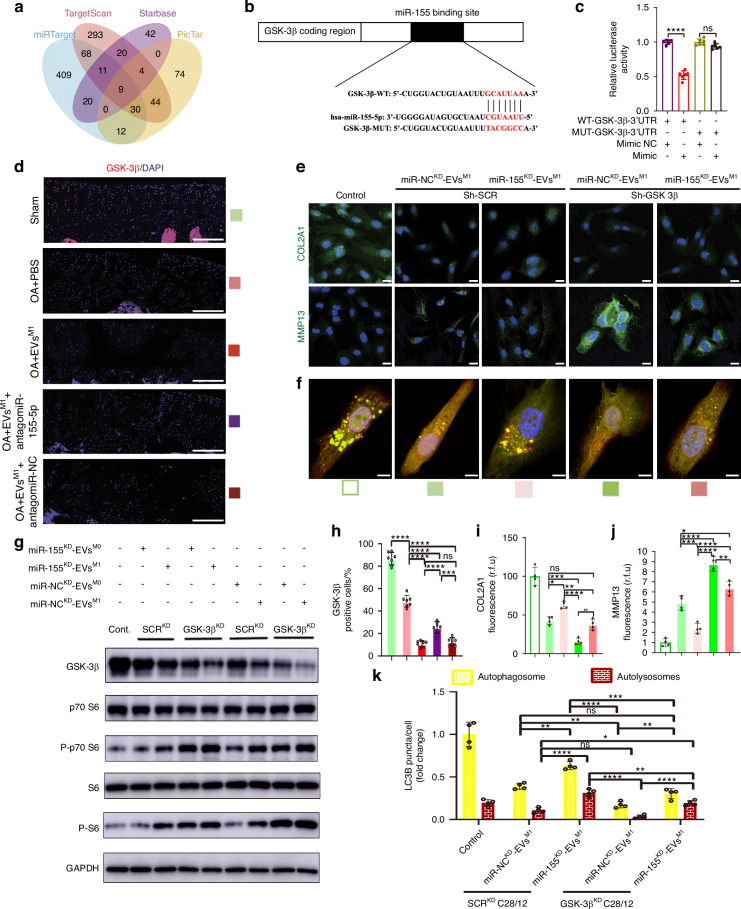
Synovial pro-inflammatory macrophages-derived EVs miR-155-5p targets GSK-3β/mTORC1 axis in OA chondrocytes. **a** The online software (TargetScan, Starbase, miRTarget, and PicTar) was used to predict the potential downstream targets of miR-155-5p. **b** Schematic representation of a predicted binding site of miR-155-5p in the 3’UTR of GSK-3β mRNA, and the mutant GSK-3β 3’UTR. **c** The luciferase activity was determined using the dual-luciferase reporter system. *n* = 6 for each group. Representative images (**d**) and quantitative analysis (**h**) of GSK-3β were assessed by IF staining in articular cartilage of sham or OA rats treated with PBS, EVs^M1^, antagomiR-155-5p and antagomiR-NC. Scale bar: 100 μm. DAPI: 4,6-diamidino-2-phenylindole. *n* = 7 for each group. Representative images (**e**) and quantitative analysis of COL2A1 (**i**) and MMP13 (**j**) were assessed by IF staining in GSK-3β^KD^/SCR^KD^ C28/I2 cells treated with EVs from miR-155-5p^KD^/miR-NC^KD^ THP-1 cell lines. Scale bar: 20 μm. *n* = 4 for each group. Representative images (**f**) and quantitative analysis (**k**) of LC3B dots per cell in GSK-3β^KD^/SCR^KD^ C28/I2 cells treated with EVs from miR-155-5p^KD^/miR-NC^KD^ THP-1 cell lines. Scale bar: 10 μm. *n* = 4 for each group. **g** Western blot showing the mTORC1 signaling pathway-related proteins, including: GSK-3β, p70 S6, P-p70 S6, S6, and P-S6 in GSK-3β^KD^/SCR^KD^ C28/I2 cells treated with EVs from miR-155-5p^KD^/miR-NC^KD^ THP-1 cell lines. One-way ANOVA &Tukey HSD post hoc test (normal distribution) and Kruskal-Wallis Test & Dunn’s test (non-normal distribution) were used for multiple comparisons. * *P* < 0.05, ** *P* < 0.01, *** *P* < 0.001, **** *P* < 0.000 1, ns not significant

To figure out the potential mechanism by which EVs miR-155-5p derived from synovial pro-inflammatory macrophages induced cartilage dysfunction and autophagy impairment, the downstream genes of miR-155-5p in cartilage have been a focus of attention. First, nine potential targets, including: GSK-3β, CEBPB, WEE1, CSNK1A1, RCN2, BACH1, ZNF644, SPRED1, CHD7, were predicted with an online database, including TargetScan, miRTarget, PicTar and Starbase prediction programs (Fig. 3a). The only four genes including GSK-3β,^34^ CEBPB,^35^ RCN2,^36^ BACH1,^37^ were reported to be relative to the progression of OA. Since microRNAs modulate gene expression by suppressing mRNA translation into proteins, we postulated that miR-155-5p aggravated cartilage destruction by inhibiting anabolic factors and autophagy-related signaling pathway molecules. Glycogen Synthase Kinase-3β (GSK-3β), which was reported to induce repairment of articular cartilage^38^ and autophagy process,^39^ was chosen to be detected further. Luciferase report tests confirmed GSK-3β 3’ UTR as a direct target of miR-155-5p. Sequences of wild-type (WT) and mutant (MUT) GSK-3β 3’ UTR were created according to predicted binding sites (Fig. 3b) and co-transfected with miR-155-5p into C28/I2 cells. The results showed that co-transfection of WT-3’ UTR of GSK-3β with miR-155-5p significantly reduced luciferase activity compared to controls, indicating specific targeting in the luciferase reporter assays. Conversely, co-transfection of MUT-3’ UTR of GSK-3β with miR-155-5p did not alter luciferase activity (Fig. 3c). Western blot analysis further corroborated these findings, demonstrating that the elevated levels of miR-155-5p correlated with decreased expression of GSK-3β. In contrast, the lower levels of miR-155-5p upregulated GSK-3β protein levels on C28/I2 cells transfected with miR-155-5p mimic or inhibitor respectively (Fig. S5A, B, C). Moreover, the antagomiR-155-5p remarkably reversed the inhibited effect of EVs^M1^-mediated GSK-3β protein in OA rats, with the negative relationship of miR-155-5 levels in articular cartilage (Fig. 3d, h). These results demonstrated that miR-155-5p directly targeted the mRNA 3’ UTR of GSK-3β to inhibit the protein levels in articular cartilage.

Next, the relevant signaling pathways of GSK-3β participated were investigated. GSK-3β inhibits the mTORC1 signaling pathway by phosphorylating TSC2.^40^ The mTORC1 signaling pathway activity, which plays a critical role in suppressing the autophagy function of chondrocytes,^41^ was determined by analyzing the phosphorylation levels of (p-p70S6K, T389) and ribosomal protein S6 (p-S6, S235/S236) in response to miR-155-5p mimic and inhibitor transfection in C28/I2 cells. Consistent with our expectations, p-p70S6K and p-S6, in ratio to their total levels, were significantly increased with miR-155-5p mimic transfection, along with the decreased protein GSK-3β. In contrast, these proteins exhibited opposite changes with miR-155-5p inhibitor transfection (Fig. S5C). Above all, we revealed that miR-155-5p upregulates the mTORC1 signaling pathway by targeting GSK-3β, then inhibits autophagy in chondrocytes and contributes to the pathogenesis of OA.

We further investigated whether synovial pro-inflammatory macrophages-derived EVs miR-155-5p could inhibit GSK-3β to exert its detrimental effect on chondrocytes. A series of loss-of-function experiments were performed in vitro. We added miR-NC^KD^-EVs and miR-155-5p^KD^-EVs into GSK-3β-knockdown C28/I2 cells. The GSK-3β expression level in GSK-3β^KD^ C28/I2 cells was significantly declined compared to the negative groups, which met the requirements of the trials (Fig. S5D). First, we found that EVs^M1-THP-1^ significantly inhibited chondrocyte function and autophagy in C28/I2 cells, compared to EVs^M0-THP-1^, no matter from miR-NC^KD^ THP-1 or miR-155-5p^KD^ THP-1 cell lines. GSK-3β knockdown exhibited the same trends as negative control in C28/I2 cells. While miR-155-5p^KD^-EVs partly reversed the impaired chondrocyte function and autophagosomes and autolysosomes formation induced by EVs^M1-THP-1^, GSK-3β knockdown could eliminate the upregulation of chondrocyte function and autophagosomal-lysomal fusion process mediated by miR-155-5p^KD^-EVs compared to miR-NC^KD^-EVs in C28/I2 cells by fluorescence intensity of COL2A1 and MMP13 (Fig. 3e, i, j) and the autophagic flux of all groups (Fig. 3f, k). Second, the mTORC1 signaling pathway activity was determined. The miR-155-5p^KD^-EVs partly reversed the increased p-p70S6K, p-S6 proteins expression levels induced by EVs^M1-THP-1^, while GSK-3β knockdown could eliminate the downregulation of p-p70S6K, p-S6 proteins mediated by miR-155-5p^KD^-EVs compared to miR-NC^KD^-EVs (Fig. 3g) by Western blotting. Taken together, we revealed that EVs miR-155-5p derived from synovial pro-inflammatory macrophages would upregulate the mTORC1 signaling pathway through targeting GSK-3β, then inhibit autophagy in chondrocytes and contribute to the pathogenesis of OA.

### FMRP selectively loads miR-155-5p into EVs in synovial pro-inflammatory macrophages

**Fig. 4:**
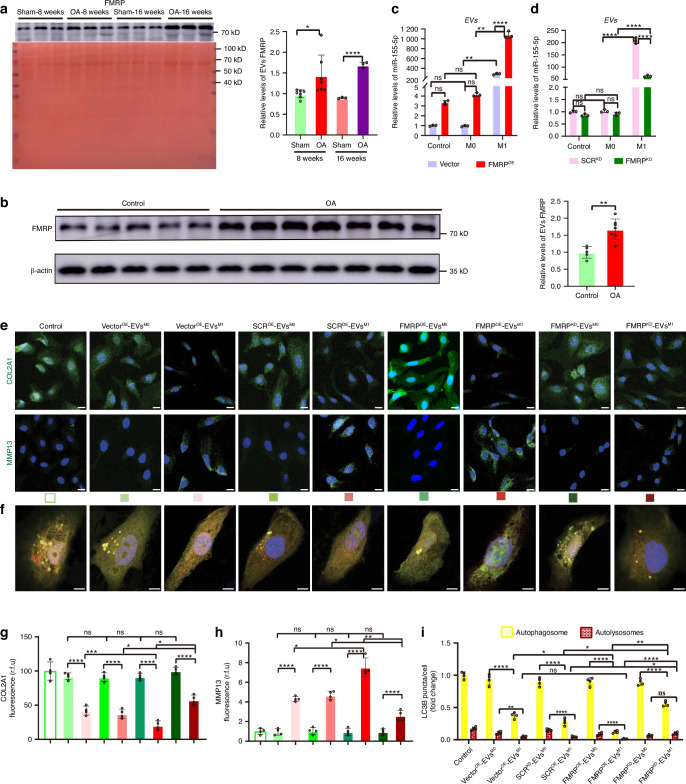
FMRP selectively loads miR-155-5p into EVs in synovial pro-inflammatory macrophages. **a** EVs were isolated from plasma of sham rats and OA rats by differential centrifugation, lysed in RIPA buffer. The protein levels of FMRP in EVs were analyzed by Western blot. Ponceau stain is shown as a loading control. **b** Western blot showing the protein levels of FMRP in plasma EVs isolated from patients with OA or healthy controls. **c** Enrichments of miR-155-5p in EVs derived from M0 and M1-polarized FMRP^OE^/Vector^OE^ THP-1 cell lines determined by qRT-PCR. **d** Enrichments of miR-155-5p in EVs derived from M0 and M1-polarized FMRP^KD^/SCR^KD^ THP-1 cell lines determined by qRT-PCR. Representative images (**e**) and quantitative analysis of COL2A1 (**g**) and MMP13 (**h**) were assessed by IF staining in C28/I2 cells treated with EVs derived from M0 and M1-polarized FMRP^OE^/Vector^OE^/FMRP^KD^/SCR^KD^ THP-1 cell lines. Scale bar: 20 μm. *n* = 4 for each group. Representative images (**f**) and quantitative analysis (**i**) of LC3 dots per cell in C28/I2 cells treated with EVs derived from M0 and M1-polarized FMRP^OE^/Vector^OE^/FMRP^KD^/SCR^KD^ THP-1 cell lines. Scale bar: 10 μm. *n* = 4 for each group. Two-tailed Student’s *t* test (normal distribution) and Mann-Whitney *U* test (non-normal distribution) were used for comparisons between the two groups. One-way ANOVA &Tukey HSD post hoc test (normal distribution), Kruskal-Wallis Test & Dunn’s test (non-normal distribution) or Two-way ANOVA & Bonferroni test were used for multiple comparisons. * *P* < 0.05, ** *P* < 0.01, *** *P* < 0.001, **** *P* < 0.000 1, ns not significant

The above results in Fig. S4B showed that the exporting of miR-155-5p into EVs could be an active process, while the levels of miR-155-5p in inflammatory macrophage is remarkably reduced compared with monocytes in the OA rats model; the specific mechanism existed to sorting miR-155-5p into EVs derived from synovial pro-inflammatory macrophages should be elucidated. The RNA-binding protein FMRP is reported to regulate EVs miR-155-5p loading through the AAUGC motif in THP-1 cells.^31^ Then the relevance of FMRP in plasma EVs and OA disease was investigated. We isolated plasma from sham rats and OA rats in the animal trials of Fig. S1A, and plasma EVs were purified according to the previous method.^42^ The levels of FMRP in plasma EVs isolated from OA rats were dramatically increased compared to sham rats. The levels of FMRP in plasma EVs significantly were increased by week 8 and onward in OA progression (Fig. 4a). Next, we isolated plasma exosomes from control human samples and OA patients, according to the previous method.^42^ The levels of FMRP in plasma EVs isolated from OA patients were significantly increased compared to control individuals. The above data indicated that enhanced levels of FMRP in plasma EVs are present in OA disease (Fig. 4b).

The manipulation of FMRP (overexpression and knockdown) was performed in THP-1 cell lines. The FMRP expression level was significantly increased in FMRP^OE^ THP-1 cell lines and decreased in FMRP^KD^ THP-1 cell lines, compared to the control groups, which met the requirements of the trials (Fig. S6). As expected, a significant augment of miR-155-5p levels was observed in EVs^M1-THP-1^ derived from FMRP^OE^ THP-1 cell lines (FMRP^OE^-EVs^M1^), compared to the negative control; meanwhile miR-155-5p levels were rapidly declined in EVs^M1-THP-1^ derived from FMRP^KD^ THP-1 cell lines (FMRP^KD^-EVs^M1^), compared to the negative control (Fig. 4c, d). Then the effect of EVs derived from FMRP^OE^ or FMRP^KD^ THP-1 cell lines on the chondrocyte function of C28/I2 cells was carried out. The findings showed that the EVs^M1-THP-1^ mediated increase of MMP13 and decrease of COL2A1 were partly reversed by FMRP knockdown and aggravated by FMRP overexpression (Fig. 4e, g, h). Moreover, the effect of EVs^THP-1^ on autophagy was determined by the autophagic flux in C28/I2 cells treated with EVs derived from FMRP^OE^ or FMRP^KD^ THP-1 cell lines. The autophagosomes and autolysosomes formation were significantly increased in C28/I2 cells treated with FMRP^KD^-EVs^M1^, while the autophagosomes formation was significantly decreased in C28/I2 cells treated with FMRP^OE^-EVs^M1^, compared to the negative control (Fig. 4f, i). The above results revealed that FMRP, which existed in synovial pro-inflammatory macrophages, plays an important role in selective sorting EVs miR-155-5p, thus destroying ECM synthesis and autophagosome formation/maturation chondrocytes in OA progression.

### Genetic knockout of miR-155-5p in synovial macrophages retards OA progression

**Fig. 5:**
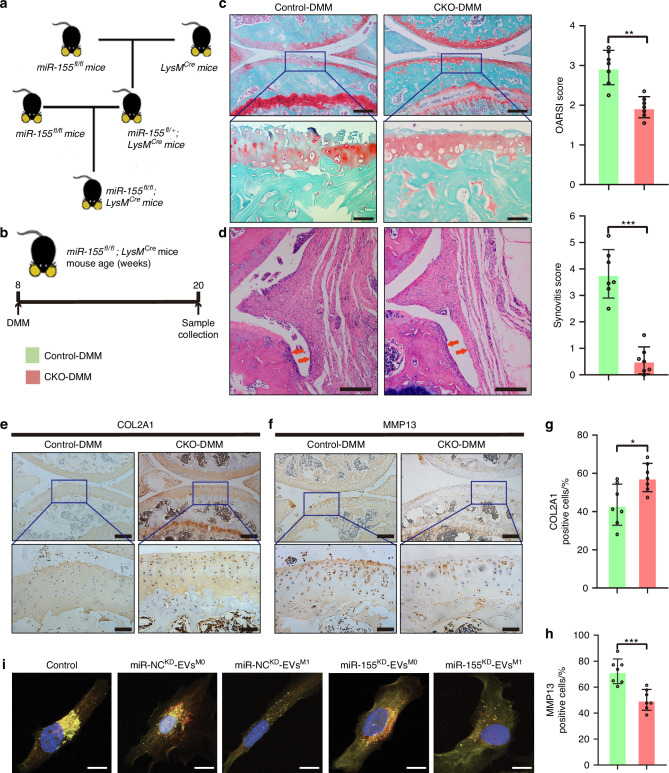
Genetic knockout of miR-155-5p in synovial macrophages retards OA progression. **a** Breeding strategy of miR-155 CKO mice. **b** A schematic diagram illustrating the experimental design. **c** SO & FG staining for keen joints in Control and CKO mice with DMM surgery (up), scale bar: 200 μm. Higher magnification images show dramatic articular cartilage changes (down), scale bar: 50 μm. (left) Quantification of OARSI score was performed using histological sections. *n* = 7 for each group. (right) **d**, Representative images (left) and quantitative analysis (right) of HE staining for the synovium in Control and CKO mice with DMM surgery, scale bar: 200 μm. *n* = 7 for each group. IHC staining of knee joint sections showing expression of COL2A1 (**e**) and MMP13 (**f**) in articular cartilage (up), scale bar: 200 μm. Higher magnification images show dramatic articular cartilage changes (down), scale bar: 50 μm. Quantification of expression of COL2A1 (**g**) and MMP13 (**h**) in articular cartilage of OA mice with IHC staining. *n* = 7 for each group. **i** Representative images of LC3 dots per cell in C28/I2 Cells treated with EVs derived from M0 and M1-polarized macrophages in miR-155-5p/miR-NC knockdown (KD) THP-1 cell lines and normal THP-1 cell (Control). *n* = 4 for each group. Scale bar: 10 μm. Two-tailed Student’s *t* test (normal distribution) and Mann-Whitney *U* test (non-normal distribution) were used for comparisons between the two groups. *P < 0.05, ** *P* < 0.01, *** *P* < 0.001, **** *P* < 0.000 1, ns not significant

To further investigate whether miR-155-5p loss in synovial macrophages impacts OA progression, myeloid-specific miR-155-5p knockout mice (*LysM*^*Cre*^; *miR-155*^*fl/fl*^) were generated (Fig. 5a) in which miR-155-5p was selectively deleted in macrophages. We performed routine genotyping of tail DNA following the instructions (Fig. S7A). Littermates carrying *miR-155*^*fl/fl*^ without Cre were used as control mice (Control). *LysM*^*Cre*^; *miR-155*^*fl/fl*^ (CKO) mice showed a 90% reduction in miR-155-5p levels in inflammatory macrophages derived from BMDMs compared with *miR-155*^*fl/fl*^ without Cre mice (Control) (Fig. S7B, C). All mice were randomized to experimental groups. At 8 weeks of age, the *LysM*^*Cre*^; *miR-155*^*fl/fl*^ (CKO) and *miR-155*^*fl/fl*^ (Control) mice were subjected to DMM operations on the right knee joints, as indicated in Fig. 5b. At 12 weeks after surgery, histomorphometric analyses were evaluated knee joint damage. Notably, CKO mice displayed less cartilage degeneration and lower OARSI scores than control mice (Fig. 5c). Similarly, the results of IHC represented that significantly increased COL2A1 expression (Fig. 5e, g) and decreased MMP13 in the cartilage of CKO mice compared to control mice during OA progression (Fig. 5f, h). Moreover, CKO mice displayed decreased synovial hyperplasia and cell infiltration levels, alongside markedly lower synovitis scores compared to control mice (Fig. 5d). Then the polarization of BMDMs to M1-like macrophages was detected using the above mice. The miR-155-5p deletion of BMDMs decreased the percentage of pro-inflammatory macrophages (Fig. S7D), which maybe demonstrate the alleviated synovitis in CKO mice. Collectively, miR-155-5p deletion in synovial macrophages alleviated OA lesions.

The effect of EVs miR-155-5p in EVs^M1-THP-1^-mediated chondrocyte dysfunction was investigated in vivo. The miR-155-5p knockdown (miR-155-5p^KD^) THP-1 cell lines were constructed, and then the EVs were isolated to treat C28/I2 cells. First, the THP-1 cells were differentiated into M0-like macrophage cells using PMA, and then LPS/ATP was used to induce M1-like polarization. The ultrafiltration was used to isolate EVs derived from M0/M1-like-THP-1 (EVs^M0-THP-1^/EVs^M1-THP-1^). Notably, the miR-155-5p levels were significantly upregulated in EVs^M1-THP-1^ and C28/I2 cells addition with EVs^M1-THP-1^, compared with the M0-like groups (Fig. S7E), suggesting that miR-155 could be transferred from THP-1 cells to C28/I2 cells through EVs. Later, the miR-155-5p knockdown of THP-1 cell lines was infected with lentivirus. As expected, reduced levels of miR-155-5p were observed in EVs^M1-THP-1^ derived from miR-155-5p^KD^ (miR-155^KD^-EVs^M1^) THP-1 cell lines, compared to the negative control (Fig. S7F). Then the effect of EVs derived from miR-155-5p^KD^ THP-1 cell lines on the chondrocyte function of C28/I2 cells were carried out. The findings showed that, the EVs^M1-THP-1^ mediated increase of MMP13 and decrease of COL2A1 were reversed by miR-155-5p knockdown (Fig. S8A, B, C). Moreover, we investigated the autophagic flux in C28/I2 cells infected with a tandem fluorescent mRFP-GFP-LC3 adenovirus (Fig. 5I). The autophagosomes and autolysosomes formation were significantly increased in C28/I2 cells treated with miR-155^KD^-EVs^M1^, compared to the negative control (Fig. S8D). On the other hand, to investigate whether the endogenous miR-155-5p in chondrocytes impacts OA progression, the cartilage-specific miR-155-5p knockout mice (*Col2a1*^*Cre*^; *miR-155*^*fl/fl*^) were generated (Fig. S7G). At 24 weeks after DMM surgery, the cartilage degeneration between *Col2a1*^*Cre*^; *miR-155*^*fl/fl*^ mice and control mice showed no significant differences in the histomorphometric analyses of knee joint damage (Fig. S7H, I).

To further preclude free miR-155-5p or other co-packaged EV components influenced by miR-155-5p levels in macrophages, the in-vitro and in-vivo experiments with EV-depleted conditioned medium from pro-inflammatory macrophages derived from bone marrow with altered miR-155-5p levels have been performed. In the in-vitro experiments, the effect of EV-depleted conditioned medium (CM) with altered miR-155-5p levels on the primary mice chondrocytes were carried out. The findings showed that, the EV-depleted CM decreased the expression of proteins COL2A1/Sox9 and increased the expression of protein MMP13, and the changes tendencies, including the increase of MMP13 and decrease of COL2A1, were promoted with the miR-155-5p mimic addition (Fig. S7J). In the in-vivo experiments, to further determine whether EVs miR-155-5p derived from synovial pro-inflammatory macrophages participated in cartilage destruction, we used EV-depleted conditioned medium (CM) and agomiR-155-5p in the CKO mice (male and female), in order to exclude the free miR-155-5p or other co-packaged EV components influenced by miR-155-5p levels in macrophages. EV-depleted CM with or without agomiR-155-5p were intra-articularly injected into the surgical-operated OA mice respectively (Fig. S7K). After 13 weeks of OA surgery, knee joints were sampled for histological analysis. AgomiR-155-5p enhanced the effect of EV-depleted CM-mediated cartilage destruction in OA rats, demonstrated with the results of OARSI scores (Fig. S7L, M). IHC analysis revealed that EV-depleted CM could decrease the expression of COL2A1, which was promoted by intra-articular injection of agomiR-155-5p (Fig. S7N, O).

The above results revealed that EVs miR-155-5p derived from synovial pro-inflammatory macrophages were vital in aggravating ECM degradation and autophagy impairment in chondrocytes.

### Engineering ADSCs-derived EVs targeting synovial pro-inflammatory macrophages represent a novel cell-free OA therapy

#### Surface modification of ADSCs-derived EVs

In the recent years, EVs derived from MSCs have emergerd as new drug delivery systems. Furthermore, EVs produced from adipose-derived stromal cells recovered by healthy donors’ liposuction, could regulate M1/M2-like macrophage polarization and ameliorate cartilage lesions/synovitis in our laboratory.^43^ The front part of this study showed that EVs miR-155-5p derived from synovial pro-inflammatory macrophages contribute to OA pathogenesis. We develop an efficient technique to realize specific delivery of antagomiR-155-5p to synovial pro-inflammatory macrophages through an engineering approach for treating OA.

First, the engineered EVs^ADSCs^-based vehicle targeting synovial pro-inflammatory macrophages was created. According to the previous reports,^44–47^ we constructed the plasmid named MAP-Lamp2b, including a glycosylation sequence (GNSTM), a pro-inflammatory macrophages-affinity peptide (MAP sequence: LPSSGAA), and a glycine-serine spacer at the N-terminus of the Lamp2b (lysosomal associated membrane glycoprotein 2b) protein. The plasmid lamp2b was constructed serving as a control without the MAP sequence. The above plasmids were respectively transfected into EVs^ADSCs^ allowing the production of control EVs (EVs^ADSCs^) and MAP-labeled EVs (MAP-EVs^ADSCs^). Then, the above EVs^ADSCs^ were isolated according to the previous differential ultracentrifuge method^48^(Fig. S9A). NTA showed that the size distribution of MAP-EVs^ADSCs^ varied from 75 to 180 nm and the main peak diameter was 130.8 nm (Fig. S9B). Then the the characters of isolated MAP-EVs^ADSCs^ were detected with TEM analysis, representing a sphere-shaped morphology and a size around 120 nm (Fig. S9C). WB analysis of the protein lysates from the ADSCs and purified EVs showed that the MAP-EVs^ADSCs^ and EVs^ADSCs^ highly expressed Alix, CD9, Tsg101 and Lamp2b, but merely expressed the endoplasmic reticulum membrane-related marker Calnexin, which only expressed in the corresponding transfected ADSCs (Fig. S9D). The above results demonstrated that the separated MAP-EVs^ADSCs^ were highly quantified to the standards for the concentration, morphology, size distribution and protein markers.

**Fig. 6:**
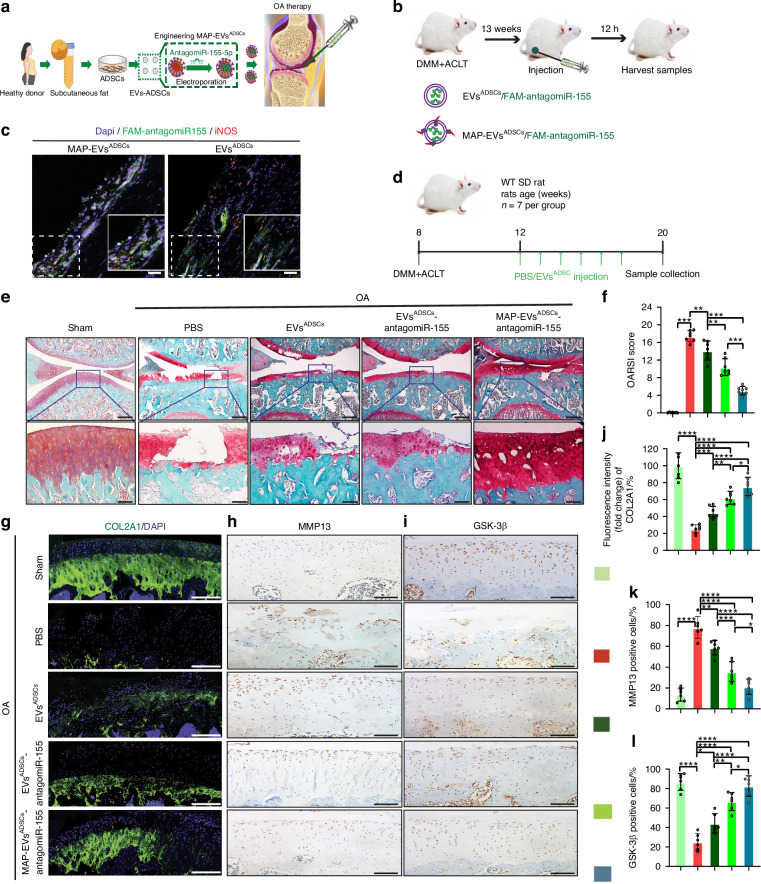
Engineering ADSCs-derived EVs promote cartilage repairment in OA model. **a** Scheme showing the design of engineering ADSCs-derived EVs. **b** Schematic illustration of the animal experimental procedure. EVs^ADSCs^ or MAP-EVs^ADSCs^ loading with FAM-antagomiR-155-5p were intra-articular injected into OA rats, and the joint samples were collected after 12 h. **c** IF staining of synovial pro-inflammatory macrophages and fluorescent images of synovium tissues treated with EVs^ADSCs^ or MAP-EVs^ADSCs^ loading with FAM-antagomiR-155-5p. scale bar: 100 μm. **d** A schematic diagram to evaluate the therapeutic efficacy of engineering EVs^ADSCs^ in OA rats model. Representative images (**e**) and quantitative analysis (**f**) of SO & FG staining in keen joints of sham or OA rats treated with PBS, EVs^ADSCs^, EVs^ADSCs^-antagomiR-155-5p and MAP-EVs^ADSCs^-antagomiR-155-5p. (up), scale bar: 500 μm. Higher magnification images show dramatic articular cartilage changes (down), scale bar: 100 μm. *n* = 7 for each group. Representative images (**g**) and quantitative analysis (**j**) of IF staining of knee joint sections showing expression of COL2A1 in articular cartilage of the aforementioned rats treated with engineering EVs. Scale bar: 100 μm. DAPI: 4,6-diamidino-2-phenylindole. *n* = 7 for each group. Representative images (**h**) and quantitative analysis (**k**) of IHC staining of knee joint sections showing expression of MMP13 in articular cartilage of the aforementioned rats treated with engineering EVs. Scale bar: 100 μm. *n* = 6 for each group. Representative images (**i**) and quantitative analysis (**l**) of IHC staining of knee joint sections showing expression of GSK-3β in articular cartilage of the aforementioned rats treated with engineering EVs. Scale bar: 100 μm. *n* = 6 for each group. One-way ANOVA &Tukey HSD post hoc test (normal distribution) and Kruskal-Wallis Test & Dunn’s test (non-normal distribution) were used for multiple comparisons. * *P* < 0.05, ** *P* < 0.01, *** *P* < 0.001, **** *P* < 0.000 1, ns not significant

Next, antagomiR-155-5p was loaded into the engineered EVs^ADSCs^ using electroporation as previously reported,^43^ and the method has been shown not to alter the endogenous contents profile of the engineered EVs.^49^ The delivery efficacy of the engineering EVs^ADSCs^ into synovial pro-inflammatory macrophages was detected in vivo (Fig. 6a). The DMM + ACLT-induced OA rats were intra-articularly administrated with EVs^ADSCs^/FAM-antagomiR-155-5p (Control group) or MAP-EVs^ADSCs^/FAM-antagomiR-155-5p. After 12 h of injection, the OA rats were killed to collect samples (Fig. 6b). The FAM-antagomiR-155-5p (green fluorescence) was largely co-localized with inflammatory macrophages (iNOS) in the MAP-EVs^ADSCs^ groups, compared to the EVs^ADSCs^ groups (Fig. 6c). Taken together, the MAP-1 peptide specifically targeted EVs^ADSCs^ to the synovial pro-inflammatory macrophages, and effectively increased the levels of antagomiR-155-5p in synoviocytes in OA animal models.

#### Engineering ADSCs-derived EVs promote cartilage repairment in OA rats model

First of all, the DMM + ACLT-induced OA rats were treated with EVs^ADSCs^/antagomiR-155-5p or MAP-EVs^ADSCs^/antagomiR-155-5p. The solution was diluted in PBS following the procedure outlined in Fig. 6d. EVs^ADSCs^ formulations were then administrated to OA rats via weekly intra-articular injections for 6 weeks. Subsequently, the rats were euthanized, and knee joint tissues were collected for comprehensive analysis. The effect of the engineering EVs^ADSCs^ on the cartilage repairement was detected. SO & FG staining results indicated significant articular cartilage damage following OA surgery, whereas the EVs^ADSCs^-treated groups showed improvement in cartilage lesions (Fig. 6e). The group injected MAP-EVs^ADSCs^/antagomiR-155-5p showed significant enhancement compared to the only EVs^ADSCs^ injected group and EVs^ADSCs^/antagomiR-155-5p injected group. The OARSI scoring outcome demonstrated that all the EVs^ADSCs^-treated groups attenuated cartilage lesions, with the MAP-EVs^ADSCs^/antagomiR-155-5p showing the most significant effects following OA surgery (Fig. 6f). Additionally, the expression of COL2A1 and MMP13 in cartilage of the joints were evaluated in OA rats. The levels of proteins COL2A1 were increased in all EVs^ADSCs^-treated groups compared to the PBS group; notably, the MAP-EVs^ADSCs^/antagomiR-155-5p exhibited the most pronounced effects post OA surgery (Fig. 6g, j). In contrast, the levels of proteins MMP13 showed the opposite trend with EVs^ADSCs^ therapy (Fig. 6h, k). Moreover, the miR-155-5p levels of synovium and cartilage were assessed in OA rats. The findings indicated a significant downregulation of miR-155-5p levels in both cartilage and synovium of OA rats compared to the sham group. Particularly in the synovium of OA rats treated with MAP-EVs^ADSCs^/antagomiR-155-5p, the decrease in miR-155-5p levels was more pronounced than in the cartilage (Fig. S9E, F). Subsequently, the expression of GSK-3β, a target gene of miR-155-5p, was assessed in articular cartilages across all groups. The results demonstrated increased levels of proteins GSK-3β in all the EVs^ADSCs^-treated groups compared to the PBS group. Specifically, the group treated with MAP-EVs^ADSCs^/antagomiR-155-5p exhibited the most notable effect following surgery, correlating with the observed change in miR-155-5p levels in articular cartilages (Fig. 6i, l). These results indicated that the engineering EVs^ADSCs^, especially MAP-EVs^ADSCs^/antagomiR-155-5p, could improve cartilage repair by inhibiting the effect of EVs miR-155-5p derived synovial pro-inflammatory macrophages through GSK-3β mediated-chondrocytes homeostasis.

#### Engineering ADSCs-derived EVs alleviate synovitis in OA rats model and human subjects

**Fig. 7:**
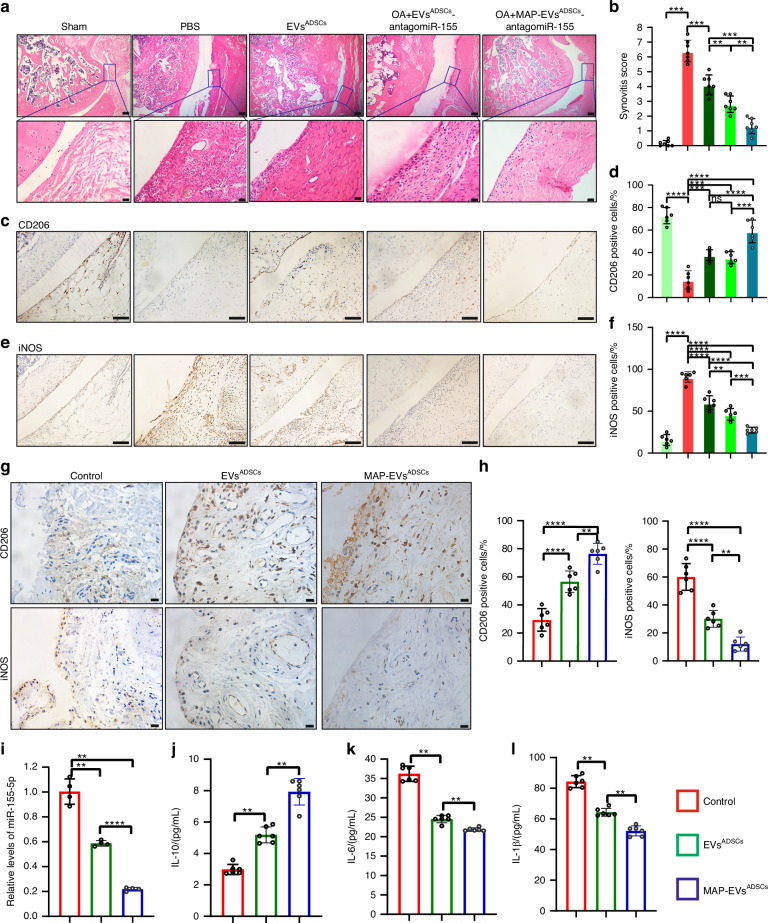
Engineering ADSCs-derived EVs targeting synovial pro-inflammatory macrophages represent a novel OA therapy. Representative images (**a**) and quantitative analysis (**b**) of HE staining in the synovium of sham or OA rats treated with PBS, EVs^ADSCs^, EVs^ADSCs^-antagomiR-155-5p and MAP-EVs^ADSCs^-antagomiR-155-5p. (up), scale bar: 200 μm. Higher magnification images show dramatic synovitis changes (down), scale bar: 20 μm. *n* = 7 for each group. Representative images (**c**) and quantitative analysis (**d**) of IHC staining of knee joint sections showing expression of CD206 in articular cartilage of the aforementioned rats treated with engineering EVs. Scale bar: 100 μm. *n* = 6 for each group. Representative images (**e**) and quantitative analysis (**f**) of IHC staining of knee joint sections showing expression of iNOS in articular cartilage of the aforementioned rats treated with engineering EVs. Scale bar: 100 μm. *n* = 6 for each group. Representative images (**g**) and quantitative analysis (**h**) of IHC staining with CD206 (*n* = 6) and iNOS (*n* = 6) in synovium from clinical OA patient treated with EVs^ADSCs^ and MAP-EVs^ADSCs^. Scale bar: 20 μm. **i** Enrichments of miR-155-5p in EVs derived from M1-polarized THP-1 cells treated with EVs^ADSCs^ and MAP-EVs^ADSCs^. *n* = 4 for each group. **j**-**l** The concentrations of IL-10, IL-6 and IL-1β in the supernatants of synovium from clinical OA patient treated with EVs^ADSCs^ and MAP-EVs^ADSCs^ using Elisa asssy. *n* = 6 for each group. One-way ANOVA &Tukey HSD post hoc test (normal distribution) and Kruskal-Wallis Test & Dunn’s test (non-normal distribution) were used for multiple comparisons. * *P* < 0.05, ** *P* < 0.01, *** *P* < 0.001, **** *P* < 0.000 1, ns not significant

Next, the effect of the engineering EVs^ADSCs^ on the synovium was detected. EVs^ADSCs^ treatment, especially MAP-EVs^ADSCs^ decreased the severity of rat OA synovial tissue, which exhibited decreased levels of synovial hyperplasia and cell infiltration, alongside markedly lower synovitis scores compared to control knees (Fig. 7a, b). Then we identified the phenotypic characterization of macrophages in rat OA synovial tissue with CD206 (M2-like macrophage marker) and iNOS (M1-like macrophage marker). In all the EVs^ADSCs^-treated groups, including notably the MAP-EVs^ADSCs^/antagomiR-155-5p, the expression of CD206 was elevated (Fig. 7c, d), whereas iNOS expression exhibited a contrasting effect (Fig. 7e, f).

Building upon the encouraging results of engineering EVs^ADSCs^ in animal OA models, we explored their translational potential in human subjects. The synovium samples from OA patients were treated with the engineering EVs^ADSCs^ in vitro. EVs^ADSCs^ treatment, especially MAP-EVs^ADSCs^ exhibited a significant downregulation in the proportion of iNOS-positive macrophages and an upregulation in the percentage of CD206-positive macrophages within the synovium tissues from OA patients (Fig. 7g, h). Furthermore, levels of anti-inflammatory and pro-inflammatory cytokines in the supernatants from clinical synovial tissue samples were assessed following EVs^ADSCs^ administration. The levels of IL-10 in the synovium tissues from OA patients was markedly increased with the engineering EVs^ADSCs^ treatment, especially MAP-EVs^ADSCs^ (Fig. 7j). EVs^ADSCs^ treatment reduced concentrations of IL-6 and IL-1β in synovial tissues from OA patients, especially MAP-EVs^ADSCs^ treatment (Fig. 7k, l). The miR-155-5p levels of EVs derived from the M1-like macrophage was detected on THP-1 cells co-cultured with the engineering EVs^ADSCs^. The outcome indicates that the engineering EVs^ADSCs^ treatment, especially MAP-EVs^ADSCs^, considerably reduced the levels of miR-155-5p on EVs derived from M1-like macrophage (Fig. 7i). Collectively, these data showed that the engineering EVs^ADSCs^ especially MAP-EVs^ADSCs^, could inhibit M1-like macrophage polarization and the section of inflammatory cytokines; aligned with our animal trial results. These findings confirmed the beneficial impact of MAP-EVs^ADSCs^ on human synovium tissues, suggesting that MAP-EVs^ADSCs^ could serve as a promising therapeutic agent for treating synovitis in OA.

These results indicated that engineering EVs^ADSCs^ especially MAP-EVs^ADSCs^, could suppress synovitis by mediating the immune microenvironment by regulating the polarization of M1/M2-like macrophages and the secretion of pro-inflammatory and anti-inflammatory cytokines.

## Discussion

The current study elucidated the mechanism of synovium-cartilage cross-talk in the early stage of OA, which induces the onset and progression of chondropathy in the clinic. This study first revealed that active transfer of synovial pro-inflammatory macrophages-derived EVs miR-155-5p could destroy the cartilage homestasis by impairing the autophagy of normal chondrocytes in the OA progression.

OA is a well-recognized chronic inflammatory condition affecting the entire joint. Synovitis, characterized by increased macrophage accumulation in the synovial lining, is widely implicated in the onset of OA pathology.^11^ The newest study employed single-cell and single-nuclei RNA sequencing of human synovial tissues to profile their cellular composition.^50^ Comparative analysis revealed that OA samples contained a significantly higher proportion of inflammatory macrophages than samples from healthy donors (HD). Despite monocyte and macrophage constituting the predominant myeloid lineage cell types in HD and OA conditions, comprising ~80% of all myeloid cells, inflammatory macrophages were notably enriched in OA samples.^50^ Moreover, in the anterior cruciate ligament rupture-based OA mouse model, synovial macrophage derived from myeloid cells underwent the most drastic increase in abundance at 7 days after surgery.^51^ Our study provides a demonstration that EVs derived from synovial pro-inflammatory macrophages migrated from BMDMs exacerbated cartilage lesions and induced the onset of OA on sham models, which could better clarify the clinical phenomena that there is a close link between early synovial activation and later structural deterioration accompanied by knees without early cartilage damage.^11^ The decreased autophagy was the key reason for cartilage degeneration caused by EVs secreted from synovial pro-inflammatory macrophages. Similar to the clinical questions, autophagy is decreased with aging articular cartilage. At the same time, not all elderly individuals are diagnosed with OA or show the typical OA pathology at autopsy, but variations in chondrocytes and ECM are inevitable and can eventually lead to overt OA.^52,53^ Autophagy is essential to maintain normal chondrocyte function and survival of the cartilage, a postmitotic tissue with an extremely slow cell rate turnover.^53^ Our study showed the critical role of autophagy in early chondropathy and the effect of autophagy on the synovium-cartilage cross-talk in the early stage of OA.

More significantly, we found many elevated EVs miRNAs derived from synovial pro-inflammatory macrophages, including let-7b-5p, miR-211-3p, miR-652-5p, miR-155-5p, miR-16-5p; and eventually demonstrated miR-155-5p is the only both upregulated in synovial pro-inflammatory macrophages derived EVs and clinic OA cartilage samples. Previous studies have found that miR-155-5p is processed from B-cell integration cluster (BIC), a non-coding transcript highly expressed in monocytes/macrophages, activated B and T cells.^54^ It has been widely reported that miR-155 is upregulated in M1-like macrophages.^53,55,56^ Furthermore, EVs miR-155 generated from macrophages was studied for transfer into cardiac fibroblasts to cause cardiac inflammation^57^ and vascular endothelial cells to worsen spinal cord damage.^56^ Several studies have identified miR-155 as an autophagy suppressor in OA progression using T/C28a2 cells,^58^ rat primary chondrocytes.^59^ Therefore, the more increased levels of miR-155-5p in synovium compared to the cartilage in the OA progression inspired us to investigate further whether the synovial pro-inflammatory macrophages could induce cartilage degeneration through EVs miR-155-5p. This study showed that antagomiR-155-5p remarkably reversed the attenuated function of EVs^M1-BMDMs^-mediated autophagy in the cartilages of the OA rats model.

Previous studies have observed that the expression of miR-155 was up-regulated in PMBC^60^ and synovial memebrane cells^61^ of rheumatoid arthritis (RA) patients, CD68^+^ macrophages in the membrane-lining layer and CD14^+^ in synovial fluid (SF)^62^; mir-155-deficient (*miR-155*^*-/-*^) mice showed reduced expression of articular pro-inflammatory cytokines, generation of B and T cells, local bone destruction in RA model.^62,63^ The potential function of miR-155 of synovial pro-inflammatory macrophages was rarely reported in OA disease. In this study, synovial macrophages-specific miR-155-5p knockout mice (*LysM*^*Cre*^^;^ *miR-155*^*f/f*^) was used. The cartilage lesion, decreased protein expression of COL2A1, and increased protein expression of MMP13 in articular cartilage were markedly dampened in this CKO mice with OA surgery. Our study presented that synovitis was alleviated in synovial macrophages-specific miR-155-5p knockout mice with OA surgery, which maybe clarified by the decreased M1-like polarization in BMDMs-derived macrophage of the above CKO mice, consistent with the previous study that M1-like polarization was inhibited in KOA SF-stimulated PMBC-derived macrophage.^64^ These results showed that miR-155 of synovial pro-inflammatory macrophages maybe regulate the polarization of infiltrating macrophages, thus alleviating synovitis, and the mechanism will be studied in the next.

Furthermore, the effect of EVs miR-155-5p on chondrocyte autophagy was clarified in the co-culture trials of EVs from miR-155-5p^KD^ THP-1 cell lines and normal C28/I2 cells. These outcomes suggest that EVs miR-155-5p derived from synovial pro-inflammatory macrophages would be the crucial molecule, which mediated the synovium-cartilage cross-talk by decreasing the autophagy of normal chondrocyte in the OA progression. A previous study showed that the change of circulating miR-155 levels in the sera of patients showed no association with disease activity in individuals with established rheumatoid arthritis (RA) or any alteration in appearance after treatment in early rheumatoid arthritis (ERA).^65^ According to previous studies in RA, circulating miR-155 in sera does not necessarily reflect levels in the synovial macrophage and OA disease activity. Our study did not investigate the circulating miR-155 levels in plasma in OA models.

Previous studies showed that miR-155-5p was involved in the OA progression through GDF6-SMAD2/3 signaling pathway,^66^ SOCS1-STAT3 signaling pathway^67^ and mTOR signaling pathway.^58,59^ Interestingly, the RNA-seq results of this study showed that the mTORC1 signaling pathway was remarkably regulated in cartilage samples treated with EVs^M1-BMDMs^. mTORC1 is a crucial regulator of autophagy initiation, and the mTORC1 signaling pathway was closely related to the severity of OA disease.^68,69^ Moreover, this study showed that EVs miR-155-5p transfer from synovial pro-inflammatory macrophages to chondrocytes could suppress protein synthesis of GSK-3β. GSK-3β, a serine/threonine protein kinase, plays an essential role in autophagy, immune response, and neuro-inflammatory injury.^70^ GSK-3β phosphorylates TSC2 and then inhibits the mTORC1 signaling pathway.^40^ Though GSK-3β was ubiquitously reported to mediate autophagy in diabetic cardiomyopathy,^71^ and Parkinson’s disease,^72^ the role of GSK-3β on the autophagy in OA diseases was not investigated. Meanwhile, few research showed GSK-3β protection in cartilage regeneration: chondrocyte-derived dECMs exhibited possibilities in cartilage regeneration in vivo through increased protein levels of GSK-3β.^73^ In this study, the negative relationship between the protein expression of GSK-3β and OA progression was observed in OA rats models; then antagomiR-155-5p remarkably reversed the inhibited effect of EVs^M1-BMDMs^-mediated GSK-3β protein in OA rats. These data first showed that the miR-155-5p can directly target the mRNA 3’ UTR of GSK-3β to inhibit the protein expression in the cartilage of the articular region of the OA model. Furthermore, we validated that synovial pro-inflammatory macrophages-induced autophagy inhibition and ECM degradation were mediated by transport of EVs miR-155 accompanied by inhibition of GSK-3β-induced mTORC1 signaling pathway inactivation. In line with these data, previous studies showed that miR-155 suppress autophagy dependently via activation of the mTOR signaling pathway^58^ or PI3K/AKT/mTOR signaling pathway^59^ in chondrocytes. These above studies demonstrated that the GSK-3β-induced mTORC1 signaling pathway inactivation accounted for the autophagy disruption in cartilage mediated by EVs miR-155-5p derived from synovial pro-inflammatory macrophages in the OA progression.

Sorting of miR-155-5p into EVs derived from inflammatory macrophages is an active process. However, the mechanism of miR-155-5p destined for export remains elusive. Our study revealed that FMRP in pro-inflammatory macrophages plays an essential role in sequestering EVs miR-155-5p, thus aggravating autophagy impairment and ECM degradation in normal chondrocytes in OA progression. Similar to the previous research, FMRP is crucial in selectively loading and secreting miR-155-containing EVs in Hela and THP-1 cells during inflammation.^31^ This study first showed that increased levels of FMRP in plasma EVs are present in OA disease, which might be suggested to serve as a potential candidate for OA disease. The deep mechanism of the increased levels of FMRP in plasma EVs, including the origin and specificity in OA progression, quantitative link between synovial macrophage EV FMRP and circulating plasma EV FMRP; would be investigated in the futher study, in order to confirm the biomarker accurately reflecting the onset and progression of OA. Besides this also needs to be further verified in a larger cohort of patients. The newest report identified FMRP as an immunoregulatory regulator for T cells in the tumor microenvironment.^74^ Whether FMRP participated in synovial pro-inflammatory macrophages polarization and synovitis will be studied in the next.

In clinical studies among individuals with established or advanced OA, candidate ailment-modifying medicines failed to indicate effectiveness. In this study, we developed engineering EVs mediated by pro-inflammatory macrophage-affinity peptide (MAP) through combining EVs^ADSCs^ therapy and miRNA therapy. On one hand, EVs^ADSCs^ targeting synovial pro-inflammatory macrophages could polarize inflammatory macrophages toward M2/M0-like macrophages thus alleviating synovitis and EVs miR-155-5p export; on the other hand, miRNA therapy-antagomiR-155-5p could impair the polarization of circulating monocyte to M1-like macrophage, pharmacologically block EVs miR-155-5p secretion derived from synovial pro-inflammatory macrophages, and inhibit the effect of EVs miR-155-5p on cartilage degeneration. In this study, the engineering EVs^ADSCs^ were intra-articularly injected 5 weeks after OA surgery, when the cartilage of OA rats exhibited evident cartilage destruction, similar to the clinic patients with established OA diseases. However, the effect of engineering EVs^ADSCs^ in the late stage of OA must be further investigated in future research. Engineering EVs^ADSCs^, especially MAP-EVs^ADSCs^, displayed a superficial effect on human synovium tissues by mediating the immune microenvironment in clinical OA samples. After all, a beneficial and effective cell-free therapy based on engineering EVs^ADSCs^ is strongly advocated for reconstructing cartilage and OA treatments.

While our findings provide important insights, some limitations should be considered. In the sex-specific research, female patients often exhibit KOA prevalence accompany with more severe pain and structural joint damage, particularly postmenopausal women with a strong hormonal influence.^75^ Besides, females tend to have higher M2 (anti-inflammatory) macrophage activity under estrogen influence, whereas males show stronger M1 (pro-inflammatory) responses.^76^ In this study, only male rats/mice were used, due to the direct comparison of OA outcome measures or interventions, which are mainly based on male mice or rats. The sex differences in OA incidence and macrophage biology may impact on the translational relevance of the engineering ADSCs-derived EVs, which need to be verified in more females and males’ patients in the further study.

In summary, we demonstrated that synovial pro-inflammatory macrophages-derived EVs destroy the cartilage homeostasis via impairing the autophagy of normal chondrocytes partly through GSK-3β-induced mTORC1 signaling pathway by the active delivery of EVs miR-155-5p in the OA progression. FMRP selectively sorted miR-155-5p into EVs derived from synovial pro-inflammatory macrophages in OA progression. Besides, this study highlighted a novel technique based on the engineering EVs^ADSCs^, especially MAP-EVs^ADSCs^, targeting synovial pro-inflammatory macrophages as a novel and effective cell-free therapy for improving of synovitis and cartilage degeneration in OA diseases in the future. These biomodified ADSCs-EVs solved the long-standing problem of balancing anti-inflammatory and tissue repair in one go, representing an efficient therapeutic tool for treating chronic aging-related OA diseases.

## Materials and methods

### Human specimens

A total of 20 patients (OA = 13), who were admitted to the hospital for total hip arthroplasty (THA), were recruited in this research by the Department of Orthopedic Surgery, the Sixth People’s Hospital of Shanghai Jiao Tong University (Shanghai, China). Control human samples were obtained from individuals with femoral neck fractures with no history of arthritic ailment (*n* = 7). The clinical features of individuals with patients are given in Table S1. Every individual gave informed agreement to utilize their clinical data for scientific research. The samples were kept in tissue storage solution (Miltenyi Biotec) and shipped immediately after operation. This investigation was authorized by the Ethics Committee of the Sixth People’s Hospital of Shanghai Jiao Tong University. The approval number were 2023-KY-033(K) and 2024-KY-270(K).

Cartilages were excised from the hip femoral condyles, and synovial tissue was obtained from the hip joint. These specimens were kept at −80 °C. Synovial tissue samples from OA patients were divided into three sections (~5 × 5 × 3 mm^3^ for each section) and were treated with 2 mL of whole medium with or without EVs from ADSCs for 72 h in 12-well culture plates. Following incubation, synovial specimens were regularly immunohistochemistry stained. 5-μm-thick slices were treated overnight at 4 °C with primary antibodies specific for CD206 (1:4 000, Abcam, ab252921), iNOS (1:100, Abcam, ab115819). The portion was stained with an HRP detection system (Servicebio).

### Animal model

#### OA rats model

Most induced OA models are done in males mice or rats, and the direct comparison of OA outcome measures or interventions are mainly based on male animals.^77^ Males animals treated with induced OA model may be more progressive, compared to the females animals.^78^ Male wild-type (WT) Sprague-Dawley (SD) rats, 6-week-old, were obtained from SLAC Laboratory Animal Co. Ltd. (Shanghai, China). Rats were housed at Tongji University Animal Unit under normal conditions (22 ± 2 °C ambient temperature, 50% ± 5% humidity and 12-h light cycle). When the rats were 8 weeks old, they were subjected to destabilization of the medial meniscus (DMM) and ACLT operations in the right knee joints to produce mechanical instability-related OA, as previously mentioned.^79^ Specifically, the anterior cruciate ligament on the right knee was transected surgically, and meanwhile the medial meniscus was destabilized by surgically dividing the medial meniscotibial ligament. For the sham operation, the right knee joint cavity was exposed after the incision of the cutaneous and muscular planes, and then closed with suture.

For the experiments in Fig. S1A, all rats undergoing DMM + ACLT operations or sham operations were arbitrarily divided into two groups: (1) sham group and (2) OA group. After 8 or 16 weeks of OA surgery, anesthesia of the rats was kept with isoflurane at each time point, the blood samples was taken in ethylenediaminetetraacetic acid tubes (BD) and the specimens were submitted to pathological investigation.

For the experiments in Fig. 1d, all rats undergoing DMM + ACLT operations or sham operations were randomly allocated into four distinct categories: (1) sham + PBS group; (2) sham + PBS-EVs^M1^ group; (3) OA + PBS group; (4) OA + PBS-EVs^M1^ group. Three weeks after the surgery, rats received several injections of 50 μL PBS or 50 μL PBS-EVs^M1^ (1 × 10^10^ particles/mL) intra-articularly throughout the next 6 weeks (twice a week). After 10 weeks of surgery, the rats’ anesthesia was kept up with isoflurane, and the specimens were submitted to pathological examination.

For the experiments in Fig. 2e, all rats undergoing DMM + ACLT operations or sham operations were arbitrarily allocated into five distinct categories: (1) sham group; (2) OA + PBS group; (3) OA + PBS-EVs^M1^ group; (4) OA + PBS-EVs^M1^/antagomiR-155-5p group; (5) OA + PBS-EVs^M1^/antagomiR-NC group. Rats were pre-injected with 50 μL antagomiR-NC or antagomiR-155-5p (5 nmol) for 8 weeks (two times a week), 1 week following an operation, and then given numerous intra-articular injections of 50 μL PBS or 50 μL EVs^M1^ (1 × 10^10^ particles/mL) for 6 weeks (two times a week). Following 10 weeks of operation, the rats’ anesthesia was kept up with isoflurane, and the specimens were submitted to pathological evaluation.

For the experiments in Fig. 6d, all rats undergoing DMM + ACLT operations or sham operations were arbitrarily allocated into five distinct categories: (1) sham group; (2) OA + PBS group; (3) OA + PBS-EVs^ADSCs^ group; (4) OA + PBS-EVs^ADSCs^-antagomiR-155-5p group; (5) OA + PBS-MAP-EVs^ADSCs^-antagomiR-NC group. Four weeks after the surgery, the rats received several intra-articular injections of 50 μL PBS or 50 μL EVs^ADSCs^ (1 × 10^10^ particles/mL) for 6 weeks (one time a week). After 12 weeks of operation, the rats’ anesthesia was kept up with isoflurane, and the specimens were sent for pathological investigation.

The Ethical Committee of Laboratory Animals Research Center, Tongji University, authorized the animal studies listed above. The authorization number is TJAA07622701. The National Institutes of Health’s Guidelines for the Care and Use of Laboratory Animals were followed for all animal-related experiments.

#### OA mice model

The C57BL/6J-miR155^em1(flox)Cya^ mice (*miR-155*^*flox/+*^) were acquired from Cyagen Biosciences Inc (Suzhou, China). The primers utilized for verifying the flox gene in mice are Forward (5′-TGGAATAAGTCACAAGGACAGTGA-3′) and Reverse (5′-CCAAGGGTTGAGAGGAGGA ATTTA-3′). The *LysM*^*Cre*^ mice were obtained from the Jackson Laboratory (Bar Harbor, ME, USA; No. 005680). Then, *miR-155*^*flox/flox*^ mice were mated with *LysM*^*Cre*^ mice to produce myeloid lineage-specific *miR-155* knockout mice (*miR-155*^*fl/fl*^; *LysM*^*Cre*^). The C57BL/6J-*Col2a1*^*Cre*^ mice were acquired from Cyagen Biosciences Inc (Suzhou, China, No. C001474). Then, *miR-155*^*flox/flox*^ mice were mated with *Col2a1*^*Cre*^ mice to produce chondrocytes-specific *miR-155* knockout mice (*miR-155*^*fl/fl*^; *Col2a1*^*Cre*^). Regular genotyping of tail DNA was conducted as per the recommendations. In the Animal Unit of Tongji University, all mice were kept on a 12-h light cycle. The DMM or sham operations of mice were conducted on the right knee joints as described above.^79^ The Ethical Committee of Laboratory Animals Research Center, Tongji University, authorized the animal research described above. The authorized number is TJAA07622103. All animal treatments were conducted in conformity with the Guidelines for the Care and Use of Laboratory Animals of the National Institutes of Health.

For the experiments in Fig. S7K, all *LysM*^*Cre*^; *miR-155* ^*fl/fl*^ (CKO) mice (male and female) undergoing DMM operations were arbitrarily allocated into three distinct categories: (1) OA + PBS group; (2) OA + CM group; (3) OA + CM + AgomiR-155-5p group. Six weeks after the surgery, the mice received several intra-articular injections of 10 μL PBS or 10 μL CM or AgomiR-155-5p (1 nmol) for 6 weeks (twice time a week). After 13 weeks of operation, the mice’ anesthesia was kept up with isoflurane, and the specimens were sent for pathological investigation.

### Cells

The primary macrophages derived from bone marrow (BMDMs) were collected and cultivated, as mentioned earlier.^80^ The cervical dislocation was used to euthanize SD rats, and the tibia and femur were both removed. The bone marrow was washed out with a 1 mL syringe and cold phosphate-buffered saline (PBS) with 1% penicillin-streptomycin (P/S). After filtering through a 70-μm strainer, 1 mL of red blood cell lysis solution was added to eliminate red blood cells (RBC) (Solarbio, China, R1010). After waiting 10 min, cells were centrifugated for 10 min at 450 × *g*, 4 °C, and the supernatant was discarded; 10 mL PBS was put into the tube and mixed. Following centrifugation, cells were rinsed two time and then were blocked with in PBS with rat Fc Block (BD, 550271) at 4 °C for 20 min. After rinsing with PBS, cells were reconstituted in the FACS buffer containing the following antibodies: PerCP/Cyanine5.5 anti-rat CD45 antibody (1 μg/mL, Biolegend, 202220), PE/Cyanine7 anti-rat CD11b/c antibody (2.5 μg/mL, Biolegend, 201818), for 30 min at at 4 °C, and then sorted with BD FACS Flow cytometry. The sorted BMDMs were resuspended in Iscove’s modified Dulbecco’s medium (Gibco, USA) comprising 10% fetal bovine serum (FBS), 1% P/S, and 20 ng/mL M-CSF (R&D System, USA). Non-adherent macrophage precursors were cultivated after 1 day, and the medium was substituted every 3 days. On the 7th day, the mature BMDMs were defined as M0 BMDMs. To promote M1-like polarization, M0 BMDMs were incubated with Lipopolysaccharide (LPS, 100 ng/mL, serotype O55:B5, Enzo), IFN-γ (20 ng/mL) for 48 h before being stimulated with 5 mmol/L ATP for a further 60 min to activate the inflammasome. Flow cytometry was utilized to determine polarization. Moreover, BMDMs-derived EVs were isolated from the supernatant for additional studies.

The isolation of primary articular chondrocytes was performed according to the study.^81^ Cartilage samples of newborn SD rats or C57 mice by first rinsing them in sterile PBS containing P/S, following by slicing. The cartilage was then enzymatically digested to recover cells using high-glucose Dulbecco’s modified Eagle medium (DMEM) (Gibco, USA) supplemented with 1% P/S and 0.2% type II collagenase (Gibco, 17101-015). The cell suspension was passed through a 70 μm cell strainer, and the obtained cells were centrifuged at 400 × *g* for 5 min. The resulting pellets, composed of primary chondrocytes, were reconstituted in DMEM/F12 medium (Gibco, USA) supplemented with 1% P/S, 10% FBS and 1% glutamine. Every other day, fresh medium was replaced. Cells from passage three were utilized. The isolation of primary synoviocytes from 8-week old SD rats was similar to the procedure of the previous research.^32^

Adipose tissue-derived MSCs (ADSCs) were isolated following previously published protocols.^82^ Subcutaneous adipose tissue was taken from healthy and younger donors via liposuction. The study protocols and ethical guidelines were approved by the People’s Liberation Army No. 85 Hospital in Shanghai, P.R. China (review serial number NO.2013/18).^83^ A written authorization was obtained from each donor participant. All procedures were conducted following the Helsinki Declaration and standard protocols. Adipose tissue weighing 500 mg was sliced into 1-mm^3^ fragments and subjected to a 45-min digestion with 0.1% collagenase I (Gibco, USA) after rinsing with PBS. Following digestion, an equal volume of complete culture medium (DMEM-F12 culture medium with 10% FBS, 1% P/S) was added, and the mixture was centrifuged at 1 000 r/min for 10 min. Cells were cultured in a complete medium containing 10 ng/mL bFGF (Stem Cell, USA) at a density of 1 × 10^6^/mL. Following that, cells were resuspended in PBS and passed through a 40 μm cell strainer. Adherent cells were passaged at a 1:3–1:4 ratio upon reach 80%–90% confluence. ADSCs up to passage 7 were used for experiments. The Human MSC examination Kit (BD Biosciences) and flow cytometry (FACSCalibur, BD, NJ, USA) were employed to characterize ADSCs surface markers. Data was analyzed using Flow Jo V10 software.

All cell lines employed in this investigation were taken from the American Type Culture Collection (ATCC). THP-1 human monocyte cells were cultured in RPMI 1640 (VivaCell Biosciences, C3010-0500), comprising 10% FBS and 1% P/S. The cells were differentiated into macrophage-like cells treated with PMA (100 ng/mL) for 48 h, and were defined as M0-like THP-1 cells. Then, the medium was substituted with an EVs-depleted serum, and the cells were maintained for a 48-h rest period. To induce M1-like polarization, M0-like THP-1 cells were pretreated with LPS (500 ng/mL, serotype O55:B5, Enzo), IFN-γ (20 ng/mL) for 48 h and then treated with 5 mmol/L ATP for an additional 60 min to stimulate the inflammasome. Flow cytometry was utilized to determine polarization. Additionally, THP-1-derived EVs were isolated from the supernatant for further investigation.

HEK293T cells were kept in DMEM supplemented with 10% FBS and 1% P/S.

Normal human chondrocytes C28/I2 cells, were grown in DMEM supplemented with 10% FBS and 1% P/S.

All cells were kept in an environment comprising 5% CO_2_ at a temperature of 37 °C.

### Plasmids construction

The packaging plasmids: pCMV-dR8.2 (Addgene plasmid #8455) and pCMV-VSV-G (Addgene plasmid #8454) were gifts from Bob Weinberg.^84^ The original pLVX-shRNA2-ZsGreen1 (No. 632179) was purchased from Clontech, and ZsGreen1 was replaced with puromycin N-acetyltransferase to obtain pLVX-shRNA2-Puro. The scrambled and *GSK-3β* or *FMR1*-targeted shRNAs were cloned into pLVX-shRNA2-ZsGreen1 or pLVX-shRNA2-Puro. The original pLVX-IRES-ZsGreen1 (No. 632187) was purchased from Clontech, and ZsGreen1 was replaced with puromycin N-acetyltransferase to obtain pLVX-IRES-Puro. The FMR1 coding region was inserted into pLVX-IRES-Puro. The construction of miR-155-5p sponge was produced according to previous researches.^85,86^ Four identical complementary miRNA antisense binding sites (AATTACGATTAGCACTATCCCCAA) and some short linkers were designed according to the previous study.^85^ The sponges’ 5’ and 3’ ends included overhangs compatible with EcolI and BamHI restriction endonucleases. The plasmid pLVX-IRES-Puro was digested and connected with the above target sequences. The relative primer sequences are shown in Table S2.

For the production of engineering EVs^ADSCs^, the original plasmid pEGFP-C1-RVG-Lamp2b presented by Prof. Matthew J. A. Wood’s lab from the University of Oxford^45^ was cloned into plasmids encoding Lamp2b, MAP-Lamp2b and MAP-EGFP-Lamp2b respectively, according to the previous report.^44^

### Lentiviral production and transduction

The production of lentivirus was designed according to standard protocols. The vectors including: pLVX-shRNA2-Puro, pLVX-shRNA2-ZsGreen1, pLVX-shGSK3β-Puro, pLVX-shFMR1-Puro and pLVX-IRES-Puro were respectively transfected into HEK293T cells with packaging plasmids using Lipo293TM (Beyotime, C0521). The supernatants of the transfected HEK293T cells including the lentivirus were collected 2 days and 3 days post transfection and filtered with a 0.45 μm filter. For the lentiviral infection of Thp-1 cells, spinoculation (1 000 × *g* for 2 h, at 37 °C, 10 ng/mL polybrene) was used. Then Thp-1 cells were plated into 6-well plates, supplemented with 4 mL fresh medium and cultured for an additional 48 h. For the lentiviral infection of C28/I2 cells, the cells were cultured with the medium of the transfected HEK293T cells for nearly 48–72 h at 37 °C.

### Extracellular vesicles and conditioned medium isolation and characterization

Total EVs from BMDMs, THP-1 cells, or ADSCs-derived cell supernatant were isolated using the differential ultracentrifuge method.

For the extraction of the EVs from BMDMs, M0-like BMDMs, and M1-like BMDMs, the cell supernatants were rinsed two times with PBS and then grown in DMEM culture medium added with 10% EVs-depleted serum (bovine EVs were depleted using overnight centrifugation at 100 000 × *g*) for 48 h. Then, the cell supernatant was obtained.

#### Extracellular vesicles

To extract the EVs from THP-1 cells and M0/M1-like THP-1 cells, the cell supernatants were rinsed two times with PBS, then seeded in RPMI 1640 medium added with 10% EVs-depleted serum for 48 h. Following that, the cell supernatant was gathered.

For the extraction of EVs^ADSCs^, ADSCs which reached 80%–90% confluence during the second passage, were grown in DMEM-F12 culture medium added with 10% EVs-depleted serum for 24 h. Subsequently, the cell supernatant was obtained.

The aforementioned cell supernatant underwent sequential centrifugation steps at 300 × *g* for 20 min, 3 000 × *g* for 20 min, and 10 000 × *g* for 30 min to remove dead cells, cell debris and apoptotic cells. Following filtration through a 0.22 μm filter, the supernatant was transferred to ultracentrifuge tubes (Beckman, 355618) and centrifuged at 100 000 × *g* for 2 h to isolate EVs. Each pellet was reconstituted in 100 μL PBS, with all procedures conducted at 4 °C. The purified EVs were either used immediately or stored at −80 °C.

The extraction of plasma EVs was purified using the previous method.^42^ The preparation of blood samples was taken within 2 h at 4 °C. First of all, the blood samples were centrifuged for 10 min at 1 690 × *g* to obtain plasma, then the plasma was centrifuged for 30 min at 10 000 × *g* to obtain platelet-free plasma. Following filtration through a 0.22 μm filter, the supernatant was transferred to ultracentrifuge tubes (Beckman, 355618) and centrifuged at 100 000 × *g* for 2 h to isolate plasma EVs. Each pellet was reconstituted in 100 μL PBS, with all procedures conducted at 4 °C. The purified plasma EVs were either used immediately or stored at −80 °C.

The particle size distribution, purity and amount of EVs were evaluated using Nanoparticle Tracking Analysis (NTA) (NanoSight 300, Malvern Instruments Ltd, UK). Specimens were diluted to the required concentration in 1 mL of PBS before being loaded into the sample chamber. Laser light was used to track and illuminated particles undergoing Brownian motion. Each specimen underwent multiple measurements, with procedural records maintained. The scattering intensity, size distribution and particle concentration were examined using the Stokes-Einstein equation. The ultrastructure of EVs was further examined using transmission electron microscopic (TEM), and images were acquired with Hitachi HT7800 electron microscope (HT-7800, Hitachi, Japan).

For co-culture investigations of chondrocytes and BMDMs-EVs, the EVs recovered from the BMDMs culture supernatant were dyed with DiI fluorescent dye via the DiI fluorescent cell linker kit (Beyotime, C1991S), followed by rinsing in PBS and ultracentrifugation (100 000 × *g* for 70 min) at 4 °C. Lastly, DiI-labeled EVs were reconstituted in PBS for the following investigation with chondrocytes. Moreover, to detect the absorption of BMDMs-EVs by the damaged articular cartilage, the EVs obtained from the BMDMs culture supernatant were dyed with DiO fluorescent stain by means of the DiO fluorescent cell linker kit (Beyotime, C1993S), accompanied by rinsing in PBS and ultracentrifuged (100 000 × *g* for 70 min) at 4 °C. At last, DiO-labeled EVs were reconstituted in PBS for the next investigation on OA model in animals.

#### Conditioned medium

The BM-derived monocytes of femurs from *LysM*^*Cre*^; *miR-155*^*fl/fl*^ (CKO) mice were separated, and the monocytes carried the markers of CD45^+^ and CD11b^+^ were sorted using flow cytometry (Fig. S7B). Then, the sorted BM-derived monocytes were induced to M1-like polarization, the conditioned medium were collected and depleted EVs using the differential ultracentrifuge method. The EV-depleted conditioned medium (from about 10^7^ cell) was prepared into lyophilized powder, then was reconstituted in 100 μL PBS.

### Flow cytometry and fluorescently stimulated cell sorting

Macrophages were characterized by the expression of markers CD45^+^, CD11b^+^, Gr1^+^, F4/80^+^. Tibias and femurs from C57BL/6 mice were processed according to the methods described in the “Cell culture” section to isolate monocytes derived from blood. Following the breakdown of red blood cells, BM cells were inhibited for 10 min at 4 °C using PBS and mouse Fc Block (BD, 553141). After washing with PBS, cells were resuspended in FACS buffer containing the following antibodies: PerCP/Cyanine5.5 anti-mouse CD45 antibody (2.5 μg/mL, Biolegend, 103132), PE/Cyanine7 anti-mouse/human CD11b antibody (2.5 μg/mL, Biolegend, 101216), BV421 anti-mouse Ly-6G/Ly-6C antibody (2.5 μg/mL, BD, 562709), Alexa Fluor 647 anti-mouse F4/80 antibody (1 μg/mL, Biolegend, 123122), PE anti-mouse CD86 (1 μg/mL, Biolegend, 105007) for 30 min at at 4 °C. Flow cytometry was conducted using a CytoFLEX S, Beckman Coulter. The analysis of the data was done with FlowJo (v.7.6.5).

### Immunofluorescence of cells

Chondrocytes were stained following a standard protocol. They underwent three brief washes in cool PBS for 5 min each. They were then fixed in either methanol or 4% paraformaldehyde for 20 min, and blocked with a Blocking Buffer for 60 min at room temperature. After that, the chondrocytes were incubated overnight at 4 °C with the proper primary antibodies, accompanied by repeated washes in PBS for 5 min each. After incubation with the corresponding fluorochrome-conjugated secondary antibody diluted in antibody dilution buffer for 1 h, chondrocytes were kept in the dark and rewashed in PBS for 5 min each. These specimens were stained with DAPI (Sigma, 32670), and washed three times for 5 min each in PBS. Representative photos were captured using a laser scanning confocal microscope (Leica, TSC SP8). The primary antibodies used were mouse anti-COL2A1 (1:1 000, Invitrogen, MA5-12789), mouse anti-MMP13 (1:1 000, Invitrogen, MA5-14238). The Alexa Fluor 488 goat anti-mouse IgG secondary antibody (1:1 000, Invitrogen, A32723) was used as for secondary reactions. Four random fields from each group were selected and utilized for statistical examination.

### Western blot

The chondrocyte and cartilage specimens were treated with the radioimmunoprecipitation assay (RIPA) lysis solution (Epizyme, PC101, China) that contained a protease suppressor cocktail (Epizyme, GRF101, China) and a phosphatase blocker cocktail (Epizyme, GRF102). For the rat plasma samples, EVs derived from plasma were purified by differential centrifugation. Then, the obtained EVs pellet was reconstituted in RIPA lysate buffer. The amount of protein was estimated via a BCA protein testing kit (Takara, T9300A), and the specimens were immediately boiled for 10 min with the addition of a loading buffer. An equal amount of protein extracts (20 μg) were put on a sodium dodecyl sulfate-polyacrylamide gel electrophoresis (SDS-PAGE) gel for electrophoresis and transferred to PVDF membranes. The PVDF membrane was then treated successively with both primary and secondary antibodies. Lastly, the increased chemiluminescence (BI, 20-500-120) was utilized to react with secondary antibodies and the photos were taken. The COL2A1-antibody (1:1 000, Abcam, ab188570), MMP13-antibody (1:1 000, Invitrogen, MA5-14238), Sox9-antibody (1:1 000, Abcam, ab185966), Beclin1-antibody (1:1 000, CST, 3495), LC3A/B-antibody (1:1 000, CST, 12741), ATG3-antibody (1:1 000, CST, 3415), ATG7-antibody (1:1 000, CST, 8558), LAMP1-antibody(1:1 000, Abcam, ab62562), GSK-3β-antibody (1: 1000, CST, 12456), p-GSK-3β-antibody (1:1 000, CST, 5558), p70S6-antibody (1:1000, CST, 9202), P-p70S6-antibody (1:1 000, CST, 9234), S6-antibody (1:1 000, CST, 2217), P-S6-antibody (1:1 000, CST, 4858), FMRP-antibody (1:1 000, CST, 4317), GAPDH-antibody (1:1 000, CST, 5174), β-actin-antibody (1:1 000, CST, 8457), anti-rabbit IgG, HRP-linked Antibody (CST, 7074), anti-mouse IgG, HRP-linked Antibody (CST, 7076) were utilized as the antibodies.

### RNA extraction, reverse transcription, quantitative real-time (RT)-polymerase chain reaction (PCR)

Total RNA was extracted from tissues or cultured cells and exosomes using Trizol reagent (Invitrogen, 15596018/10296028). For mRNA qRT-PCR, the first-strand cDNA was synthesized with HiScript^®^ III 1st Strand cDNA Synthesis Kit (Vazyme, R312-02, China). The relative expression of mRNA was normalized to that of GAPDH (mRNA control) using the 2^−^^ΔΔCt^ method. For miRNA qRT-PCR, miRNA was converted to cDNA using the specific stem-loop primer with HiScript^®^ III 1st Strand cDNA Synthesis Kit (Vazyme, R312-02, China). The qRT-PCR assay was performed on a light cycler (Roche, Basel, Switzerland) using ChamQ^TM^ Universal SYBR^®^ qPCR Master Mix Kit (Vazyme, Q711-03). The relative levels of miRNA were calculated by the 2^−^^ΔΔCt^ method with U6 small nuclear RNA (U6 snRNA; miRNA control in cells and tissues), *Caenorhabditis elegans* miR-39-3p (cel-miR-39-3p; miRNA spike control in exosomes and different tissues), according to the the previous study.^87^ Primer sequences are shown in Table S2.

### Sequencing of EVs miRNA derived from M0 BMDMs and M1 BMDMs in rats model

Total RNAs of EVs^BMDMs^ were isolated as previously described and used for miRNA sequencing. The Agilent Rat miRNA Microarray, Release 21.0 (8*15 K, Design ID: 070154) investigation and data assessment were performed by OE Biotechnology Co., Ltd. (Shanghai, China). The microarray has 758 probes for mature miRNAs of rats.

The NanoDrop ND-2000 was used to quantify total RNA (Thermo Scientific), while Agilent Bioanalyzer 2100 (Agilent Technologies) was used to evaluate RNA integrity. The specimen labeling, microarray hybridization, and rinsing were conducted with the manufacturer’s standard methods. RNA was dephosphorylated and denatured, followed by labeling with Cyanine-3-CTP. The labeled RNAs were purified and subsequently hybridized onto a microarray. The arrays were scanned using the Agilent Scanner G2505C (Agilent Technologies) after thorough cleaning.

Feature Extraction software (version 10.7.1.1, Agilent Technologies) was used to acquire raw data. Genespring software (version 14.8, Agilent Technologies) was employed for the basic assessment. First, the raw data was processed using the quantile technique. Probes with every specimen in one group tagged “Detected” were selected for further data processing. Differentially appeared miRNAs were subsequently recognized based on fold change, and the *P* value was calculated via the *t*-test. The criteria for increased or decreased regulated miRNAs was a fold change of at least ≥2.0 and *P* value of less than ≤0.05.

### Tissues RNA sequencing

Cartilage samples from the control group (OA + PBS) and EVs^M1^ treated group (OA + EVs^M1^) rats were collected (*n* = 3/group). The entire RNA was then isolated, as previously reported.^43^ The RNA libraries were sequenced by LC Bio Technology CO., Ltd (Hangzhou, China) by means of the illumina Novaseq^TM^ 6000 platform. The bioinformatics examination, comprising genes differentially expressed (DEGs), KEGG and GSEA enrichment, was performed by means of the OmicStudio instrument at https://www.omicstudio.cn/tool. The heatmap was produced using R (https://www.r-project.org/) on the OmicStudio platform (https://www.omicstudio.cn/tool).

### Transmission electron microscopy (TEM) examination for animal cartilages

After the decalcification of the tissues, TEM analysis was conducted. Cartilage samples were incubated in 2.5% glutaraldehyde for at least 4 h at ambient temperature, followed by four rinses of 15 min with 0.1 mol/L phosphate buffer. Subsequently, samples were dehydrated using increasing concentrations of acetone, post-fixed with 1% Osmium tetroxide for 1–2 h at normal temperature, and inserted in Epon 812 resin. The materials were then sectioned into 70 nm thick slices and imaged using the JEOL JEM-1230 electron microscope.

### Histological analysis

After fixation in 4% paraformaldehyde for 24 h, the entire knee joints were decalcified at pH 7.4 using 0.5 mol/L EDTA, and subsequently inserted in paraffin. Slices of 5 μm thickness were cut for staining with Safranin O/Fast Green (SO & FG) (Solarbio, G1371) and hematoxylin and eosin (H&E) (Thermo Fisher, 7211 & 7111). The OARSI scoring system was used for cartilage destruction in SO & FG-stained areas in the medial joints, including medial femoral condyles and the medial tibial plateau. To grade OA severity of mice, 0–6 subjective grading system was performed according to the previous methods.^88^ To assess OA severity of rats, the score of combined grade (0–6 grade) and stage (0–4 stage) were recommended based on the previous reports.^89,90^ The evaluation of synovial membranes features- enlargement of the synovial lining cell layer, density of the resident cell, and inflammatory infiltrate- was conducted using H&E-stained slides. Changes were assessed using a four-point scale: none (score: 0), slight (score: 1), moderate (score: 2), and severe (score: 3).^29^ Two blinded and independent observers evaluated each parameter, and the average score was employed for statistical analysis.

### Immunohistochemistry and immunofluorescence examination

For immunohistochemistry staining, the 5-μm-thick slices were treated overnight at 4 °C with primary antibodies appropriate for MMP13 (1:500, Proteintech, 18615-1-AP), COL2A1 (1:200, Servicebio, GB11021), GSK-3β (1:1 000, CST, 12456), CD206 (0.1 μg/mL, Abcam, ab64693), iNOS (1:1 000, Abcam, ab283655). The slices were stained with an HRP detection system (Servicebio), and hematoxylin was employed to counterstain them.

For immunofluorescence staining, the portions were inhibited with a blocking buffer [1 × PBS (BI, 02-024-1ACS)/5% normal serum (Jackson lab, 005-000-121, USA)/0.3% Triton™X-100 (Sangon Biotech, A110694, Shanghai, China)] for 60 min at ambient temperature. Then, the specimens were kept overnight at 4 °C with primary antibodies appropriate for COL2A1 (1:1 000, Invitrogen, MA5-12789), mouse anti-MMP13 (1:1 000, Invitrogen, MA5-14238), GSK-3β (1:1 000, CST, 12456), F4/80 (1:100, Santa Cruz, sc-377009), iNOS (1:1 000, Abcam, ab210823), CD206 (1:1 000, Abcam, ab64693). After washing, the slices were incubated with secondary antibodies for 1 h at room temperature. The Alexa Fluor 488 goat anti-mouse IgG secondary antibody (1:1 000, Invitrogen, A32723) and Alexa Fluor 594 goat anti-rabbit IgG secondary antibody (1:1 000, Invitrogen, A32754) were used. The representative photos were taken via a laser scanning confocal microscope (Leica, TSC SP8). For each joint, the proportion of positive cells was calculated in 3–5 fields, and the median percentage was typical for each mouse. The quantitative analysis of IF staining was conducted in a double-blinded way.

### The co-culture assay

The THP-1 cells were resuspended at a density of 1 × 10^6^ cells/mL well (2 mL) and seeded in the lower chambers (6-well migration chambers, 0.4 μm pore membrane, 83.3930.041, SARSTEDT, Germany), then treated into M1-like THP-1 cells with the previous method. The upper chambers were seeded with ADSCs of 1 × 10^5^ cells/well in 6-well plates. After 72 h co-culture, the supernatant of the lower cells was used to isolate EVs using the differential ultracentrifuge method. Then, the EVs were analyzed for miRNA levels with qRT-PCR detection.

### Autophagic flux assessment

The mRFP-GFP-LC3 adenoviral vector (HB-AP2100001) was obtained from HANBIO. This vector exploits the pH difference between neutral autophagosome and acidic autolysosome to generate fluorescence, with red or red/green (yellow) fluorescence facilitating the tracking of autophagic flux. C28/I2 cells were seeded on coverslips in 24-well plates and treated with co-culture with CM^M1^/CM^M0^ or EVs^M1-THP1^/CM^M0-THP1^ or miR-155^KD^-EVs^M0/M1^/miR-NC^KD^-EVs^M0/M1^ or FRMP^KD^-EVs^M0/M1^/SCR^KD^-EVs^M0/M1^ or FRMP^OE^-EVs^M0/M1^/Vector^OE^-EVs^M0/M1^ for 48 h, up to the need of per experimental requirements to study autophagic flux in the cells. Subsequently, the mRFP-GFP-LC3 adenovirus was applied to the cells for 24 h. Following this, cells on coverslips were rinsed with cool PBS, fixed in 4% PFA, and dyed with DAPI for immunostaining of the nuclear. Images were captured using a laser scanning confocal microscope (Leica, TSC SP8). At least four images of mRFP-GFP-LC3-transfected cells were analyzed for each condition using a fluorescence microscope, and the percentage of transfected cells displaying mRFP-GFP-LC3 puncta was quantified to assess autophagosomes or autolysosomes formation.

### Assay using a dual-Luciferase reporter

The onlined bioinformatics tool miRDB was employed to identify target genes and potential binding sites for miR-155-5p and GSK-3β. Wild-type (WT GSK-3β) and mutant GSK-3β dual-luciferase reporter vectors (MUT GSK-3β) were separately constructed and co-transfected into C28/I2 cells along with miR-155-5p mimic and the negative controls. Reporter assays were conducted following the supplier direction for the dual luciferase activity detection kit (Promega, E1910, USA), using a miRNA control as a negative control. Renilla luciferase activity was normalized to firefly luciferase activity and presented as a percentage relative to the control. The OD absorbance were measured using microplate reader (SpectraMax M5, Molecular Devices, SanJose, USA).

### Elisa analysis

To quantify the levels of interleukin-10 (IL-10), interleukin-6 (IL-6), tumor necrosis factor-alpha (TNF-α) and interleukin1β (IL-1β) in the rat serum, IL-10, IL-6 and IL-1β in the supernatants from clinical synovial tissue samples co-cultured with EVs^ADSCs^, ELISA analysis with ELISA kits (Elabscience, Wuhan, China) were used following manufacturer recommendations. IL-10, IL-6, TNF-α, and IL-1β concentrations were calculated in pg/mL.

### Statistical analysis

The number of samples for every experiment was established based on our previous experience. Different investigators supervised each of the experiments. All in vitro studies were replicated at least three times separately, and all in vivo tests were carried out on at least six physiologically independent animals from each group. All attempts to replicate the experiments were effective. GraphPad Prism 8.0 was employed for all statistical examinations (GraphPad Software Inc., La Jolla, CA, USA). Two-tailed Student’s *t* test (normal distribution) and Mann-Whitney *U* test (non-normal distribution) were used for comparisons between the two groups. One-way ANOVA & Tukey HSD post hoc test (normal distribution), Kruskal-Wallis Test & Dunn’s test (non-normal distribution) or Two-way ANOVA & Bonferroni test were used for multiple comparisons. All OARSI scoring were analyzed using non-parametric tests. Data are reported as the mean ± standard error (SEM), and *P* < 0.05 is considered statistically significant. *P < 0.05, **P < 0.01, ***P < 0.001, ****P < 0.000 1, ns not significant.

### Ethics approval and consent to participate

All animal experiments in the current study were approved by the ethics and technological committees of Tongji University. The approval number was TJAA07622701, TJAA07622103.

The clinical OA samples was obtained by total hip joint replacement surgeries. The clinical control samples was obtained from the individuals with femoral neck fracture with no history of arthritic diseases. All patients provided informed consent to use their clinical information for scientific research. This study was approved by the Ethics Committee of the Sixth People’s Hospital of Shanghai Jiao Tong University. The approval number was 2023-KY-033(K) and 2024-KY-270(K). The subcutaneous adipose tissue was obtained by liposuction from young healthy donors. The methods and guidelines were approved by the People’s Liberation Army No. 85 Hospital, Shanghai, P.R. China (review serial number NO.2013/18). All donors provided written informed permission.

## Supplementary information

Data-Figure 1

Data-Figure 2

Data-Figure 3

Data-Figure 4

Data-Figure 5

Data-Figure 6

Data-Figure 7

Data-Supplementary Figures S1–S9

Figure S1

Figure S2

Figure S3

Figure S4

Figure S5

Figure S6

Figure S7

Figure S8

Figure S9

Supplementary Table1

Supplementary Table2

Supplementary information

## Data Availability

All data supporting the findings of this study are available within the article and its Extended Data files or are available from the corresponding author upon reasonable request. The publicly available RNA-Seq data and miRNA-Seq data used in this study are available in the GEO database with GEO accession number of GSE 278832 and GES 278392.
