# Supplementary figures and images for: Synovial inflammatory macrophage-derived extracellular vesicles exacerbate cartilage lesions with a FMRP-selectively sorted manner in osteoarthritis
Source: Bone Res. 2026 Feb 17;14:26. doi: 10.1038/s41413-025-00502-4 (PMC12913794; doi:10.1038/s41413-025-00502-4)

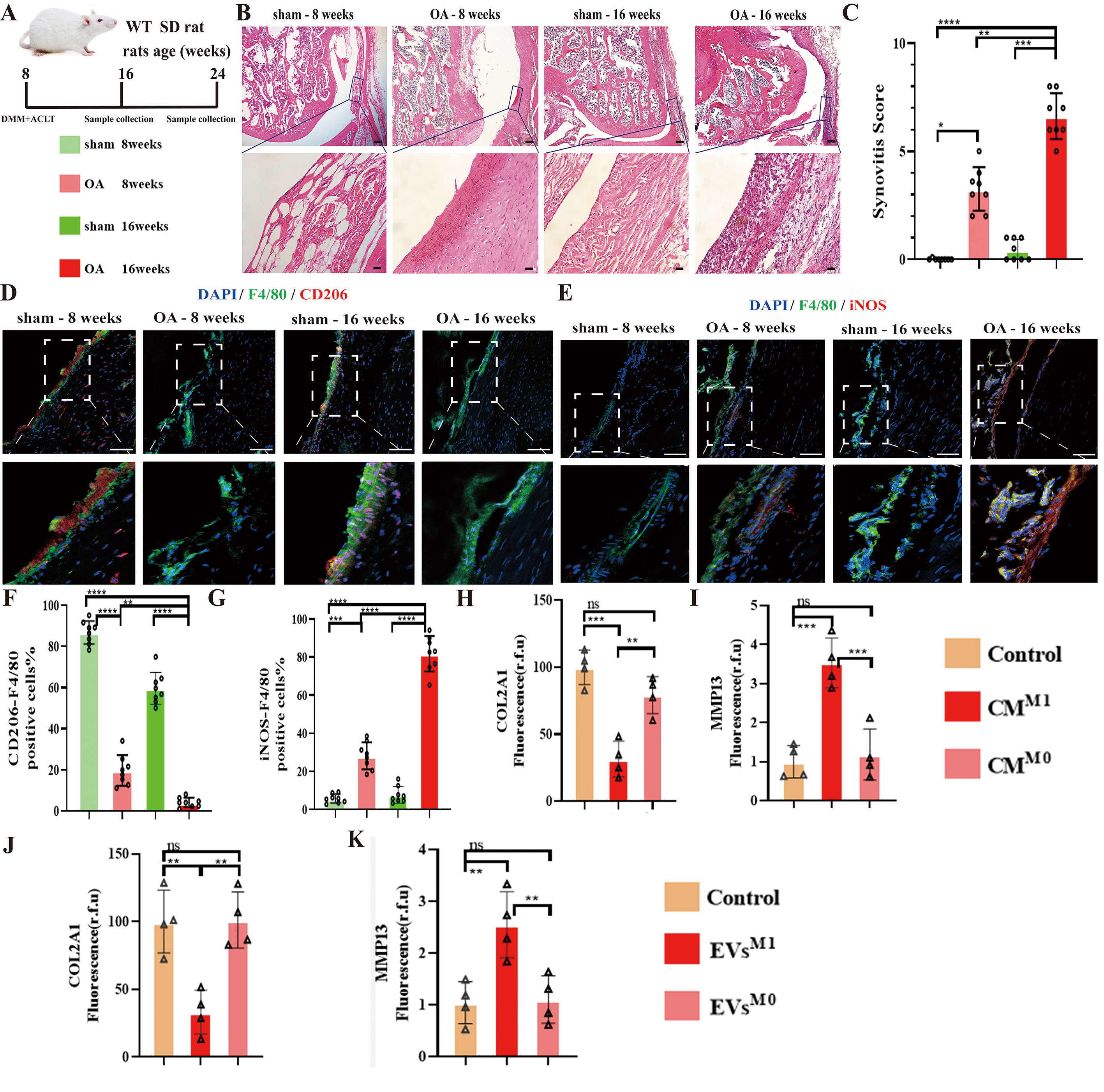

Supplement: Supplementary file 9 — Figure S1 [file 41413_2025_502_MOESM9_ESM.tif]

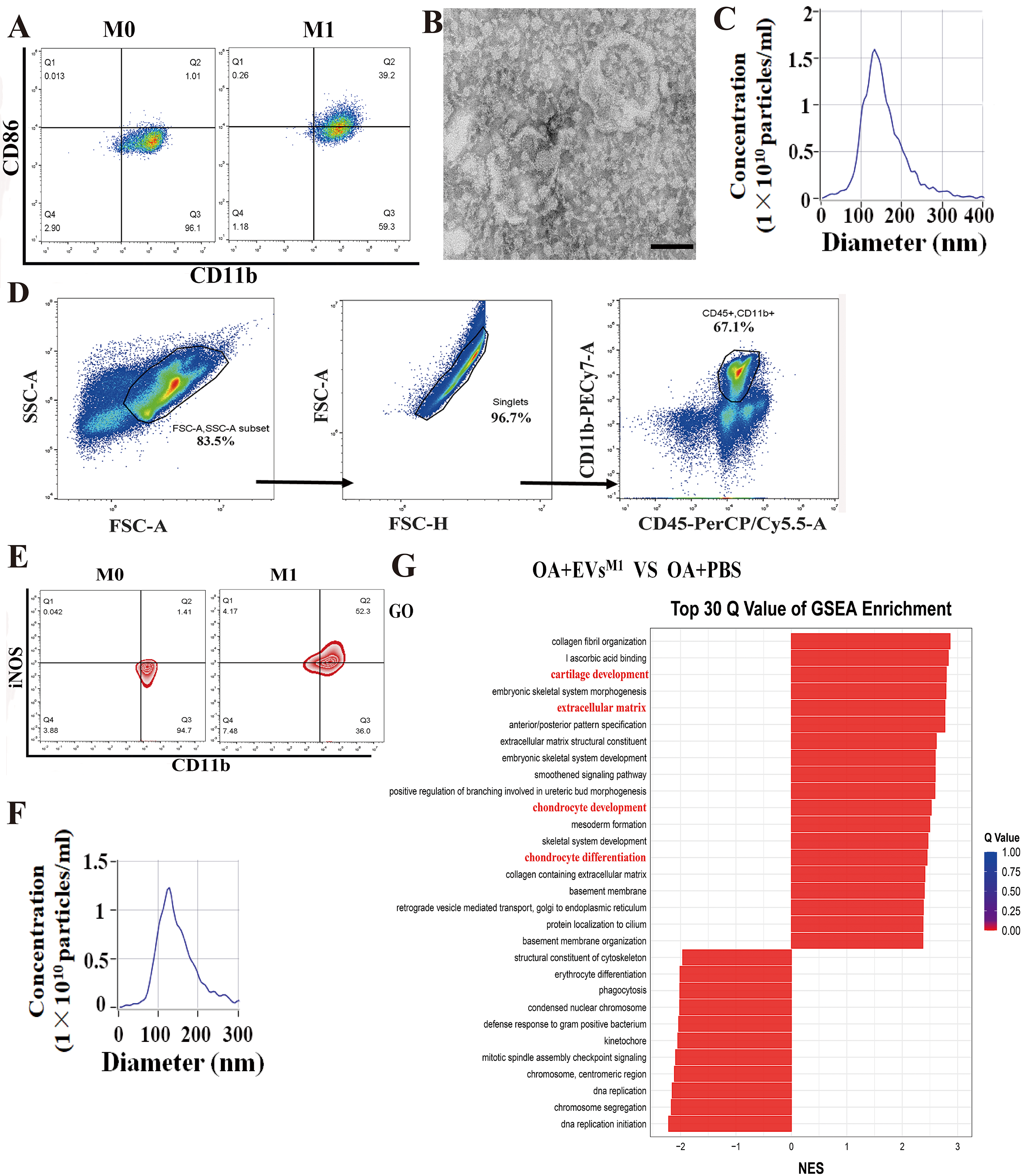

Supplement: Supplementary file 10 — Figure S2 [file 41413_2025_502_MOESM10_ESM.tif]

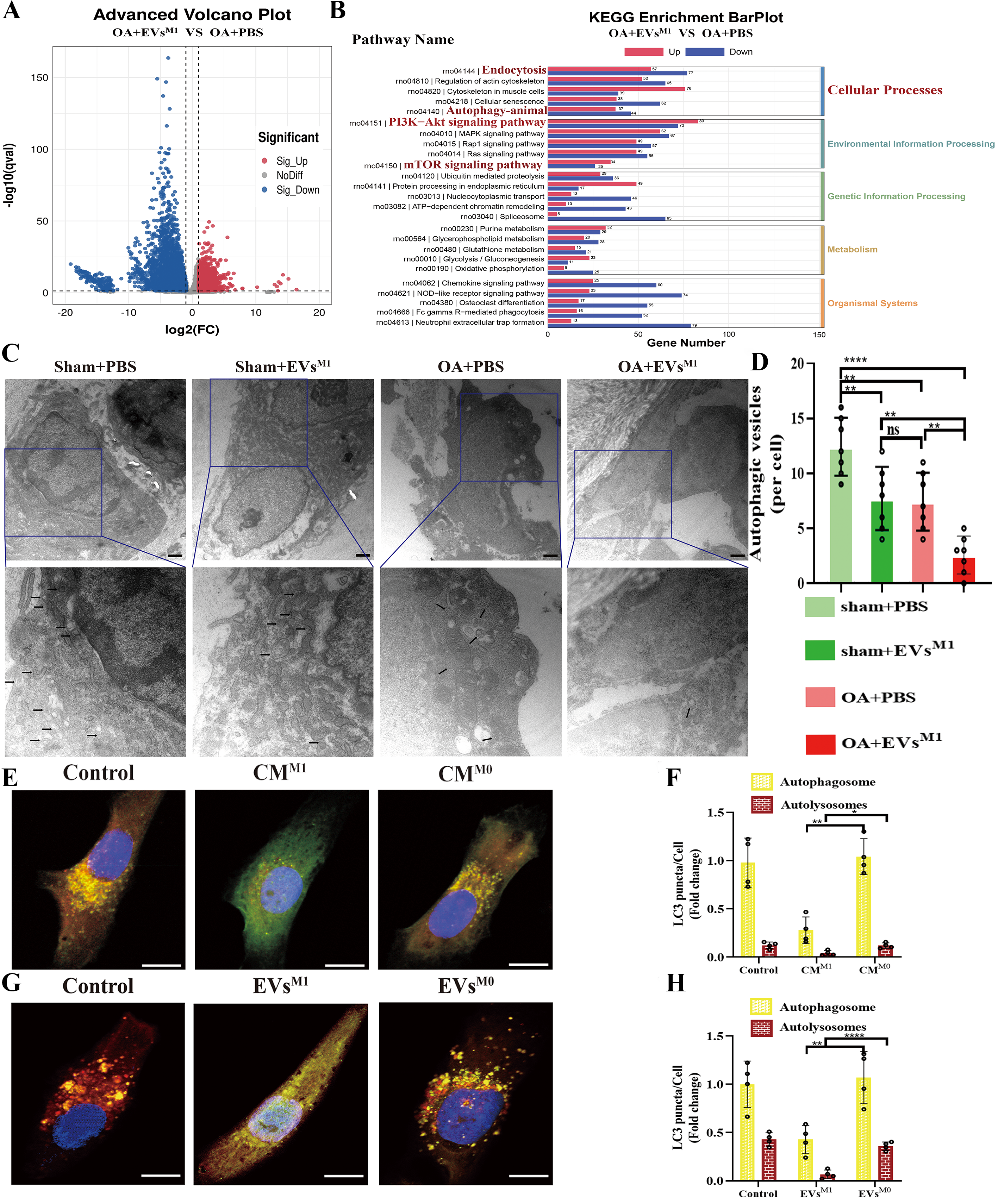

Supplement: Supplementary file 11 — Figure S3 [file 41413_2025_502_MOESM11_ESM.tif]

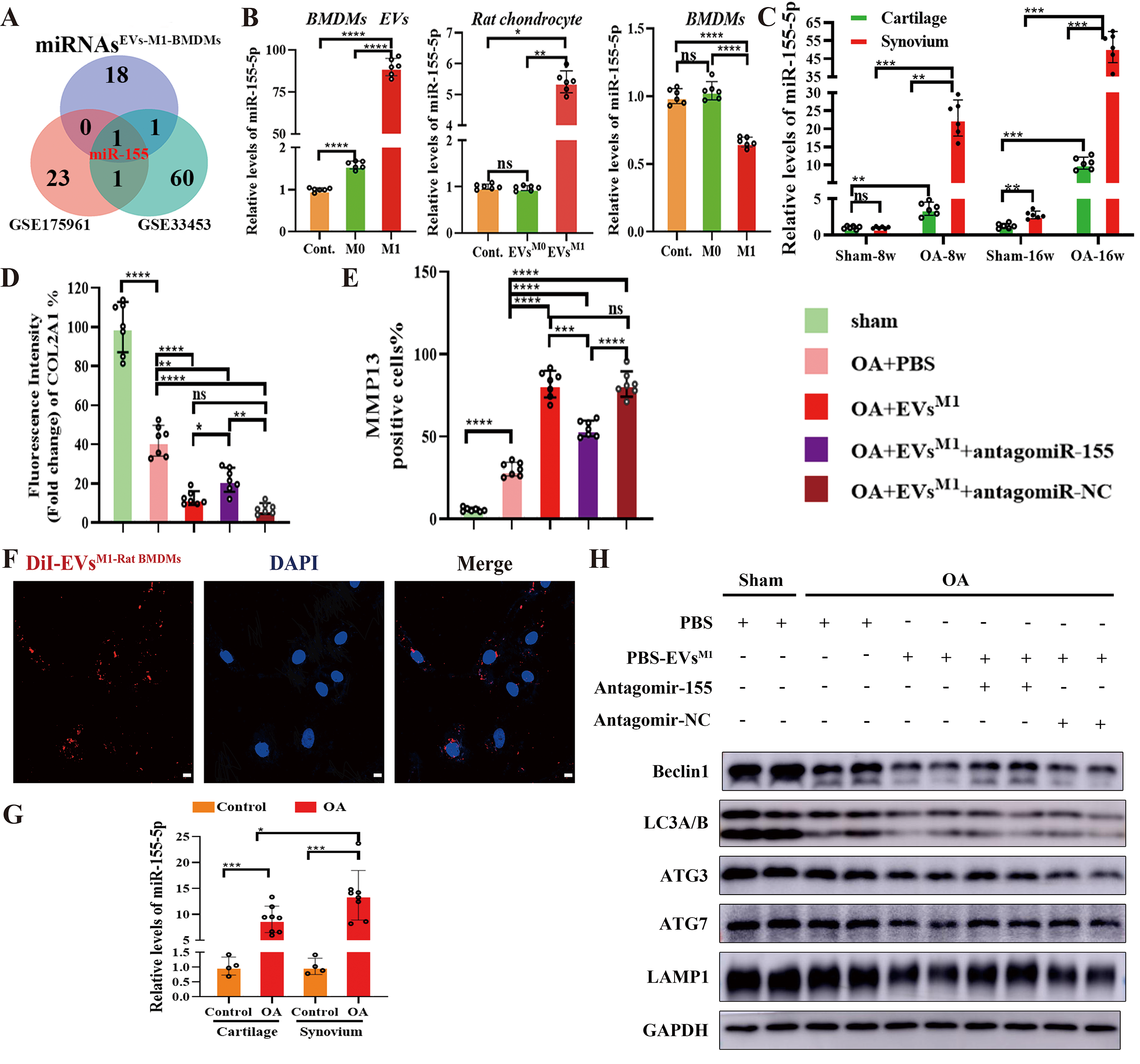

Supplement: Supplementary file 12 — Figure S4 [file 41413_2025_502_MOESM12_ESM.tif]

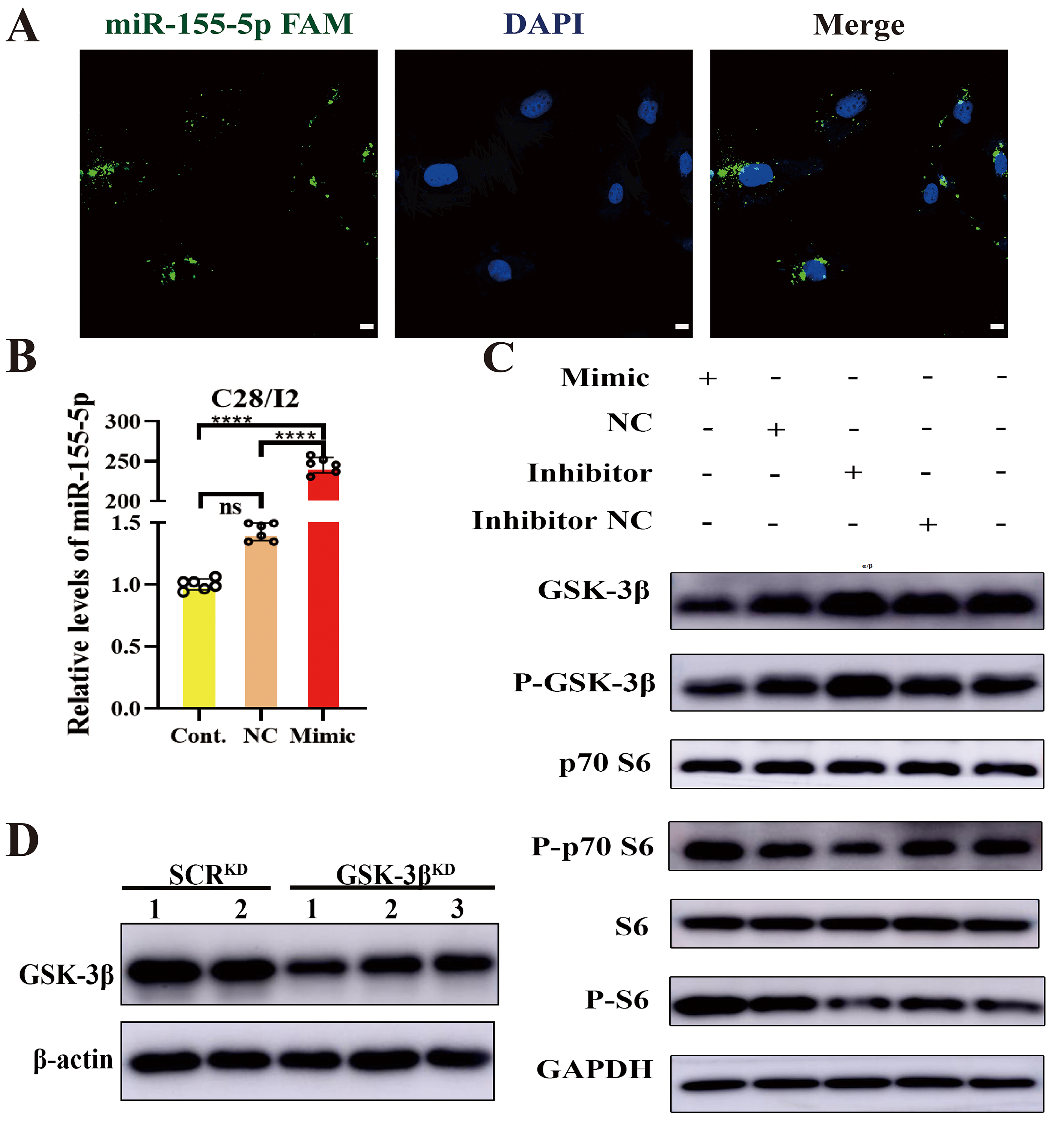

Supplement: Supplementary file 13 — Figure S5 [file 41413_2025_502_MOESM13_ESM.tif]

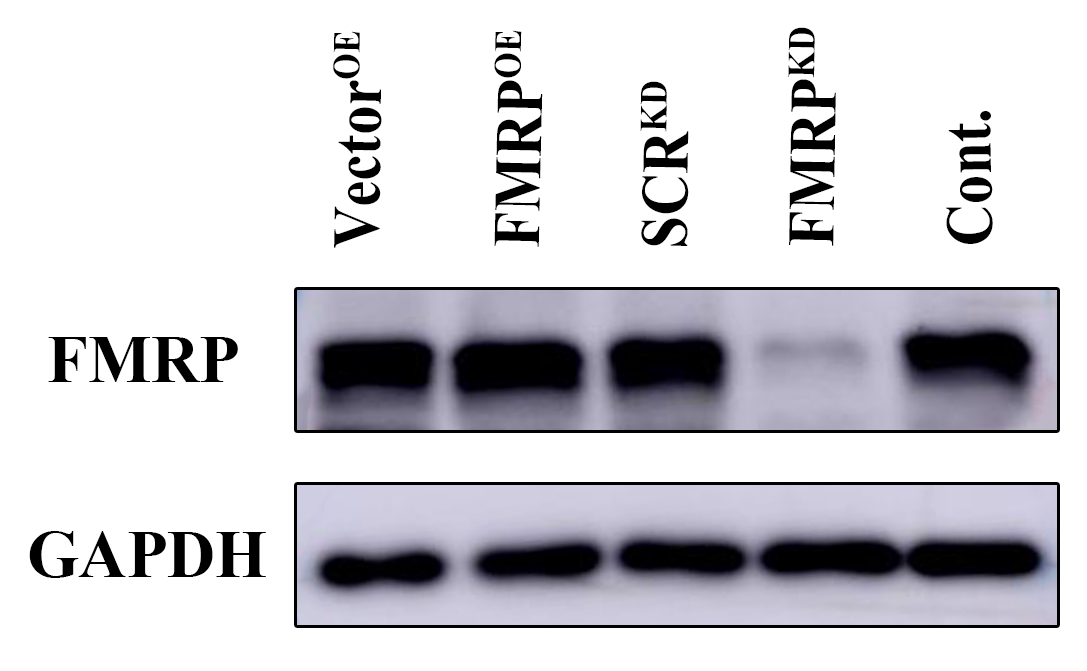

Supplement: Supplementary file 14 — Figure S6 [file 41413_2025_502_MOESM14_ESM.tif]

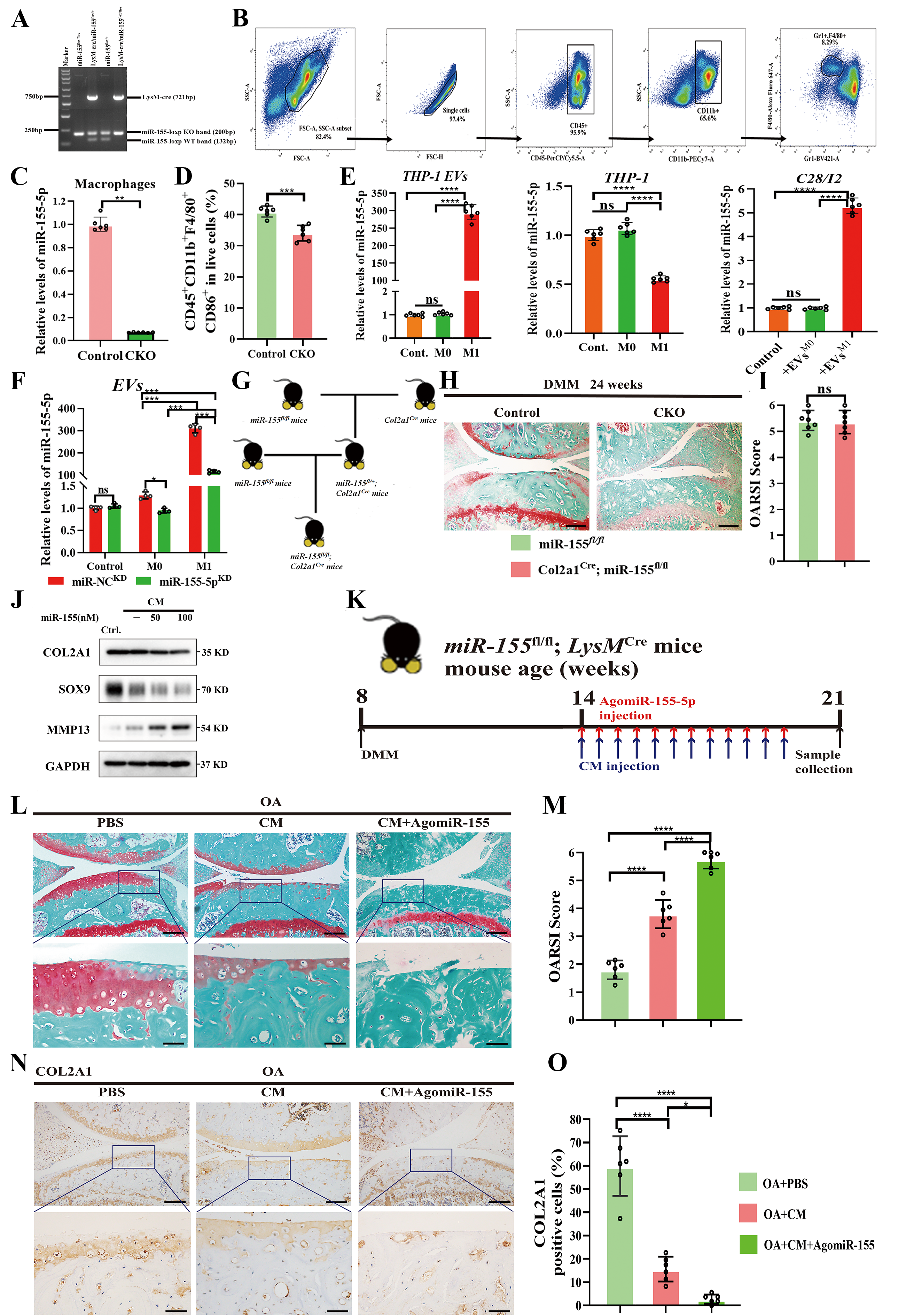

Supplement: Supplementary file 15 — Figure S7 [file 41413_2025_502_MOESM15_ESM.tif]

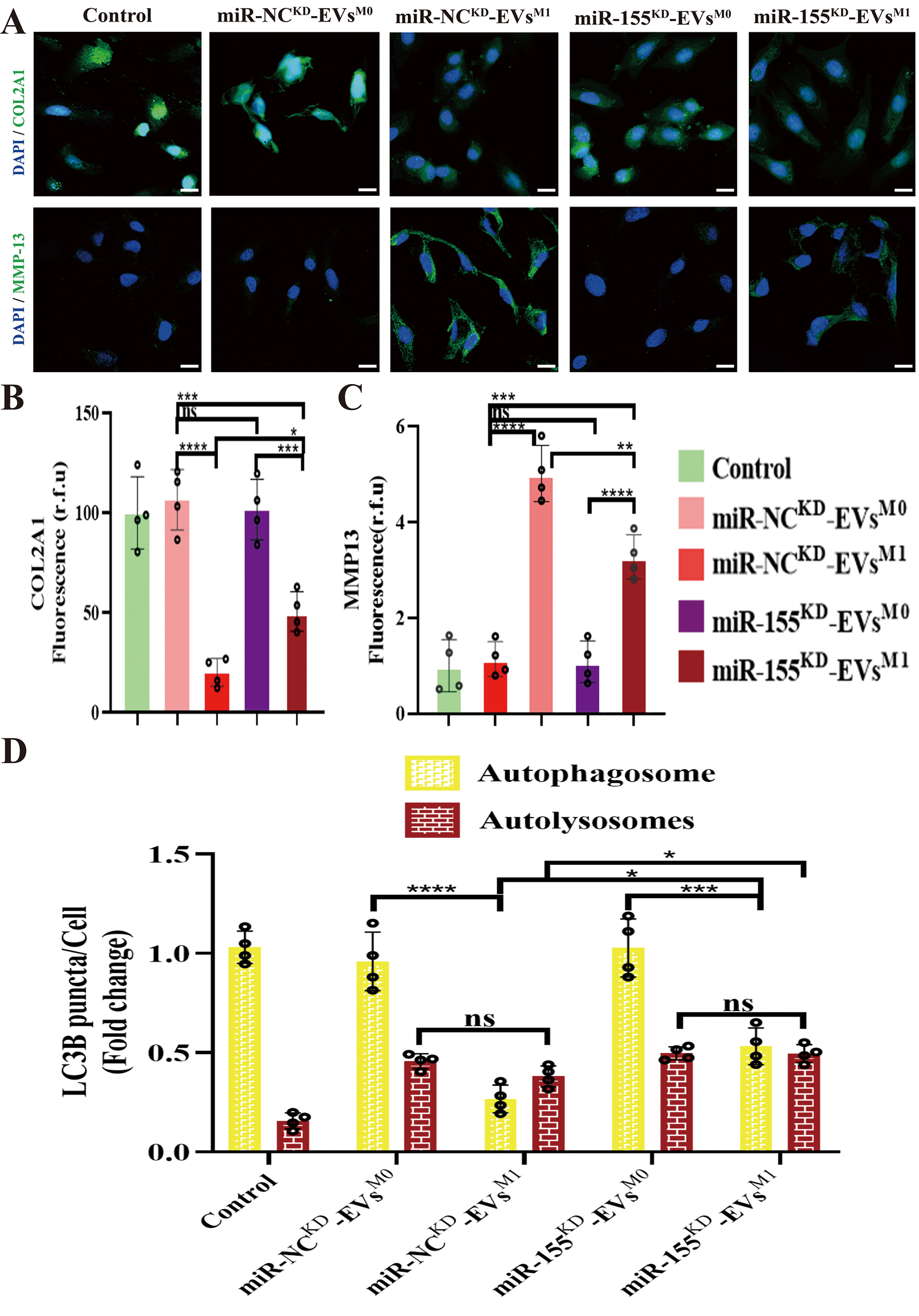

Supplement: Supplementary file 16 — Figure S8 [file 41413_2025_502_MOESM16_ESM.tif]

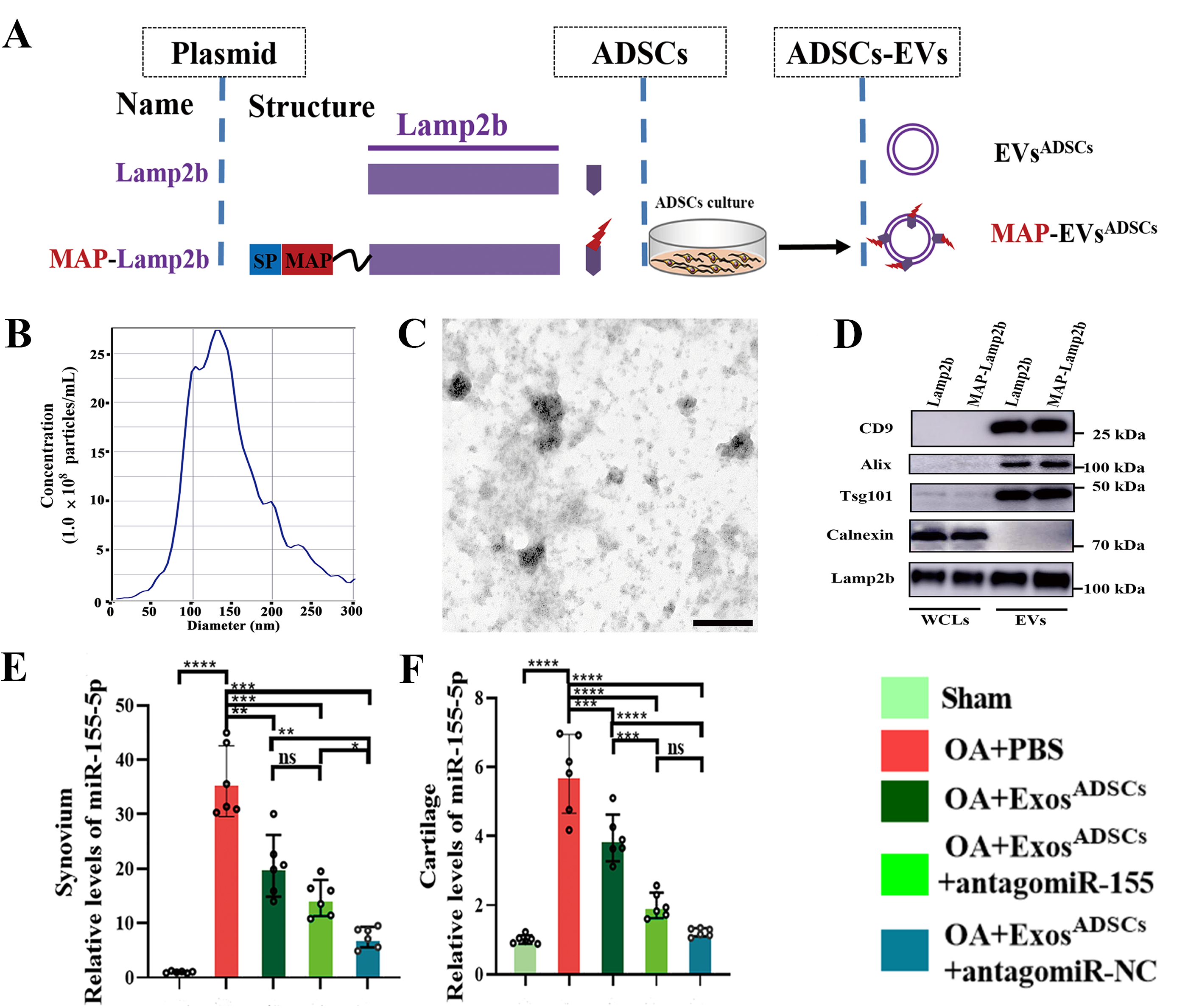

Supplement: Supplementary file 17 — Figure S9 [file 41413_2025_502_MOESM17_ESM.tif]
