# Supplementary material for: Synovial inflammatory macrophage-derived extracellular vesicles exacerbate cartilage lesions with a FMRP-selectively sorted manner in osteoarthritis
Source: Bone Res. 2026 Feb 17;14:26. doi: 10.1038/s41413-025-00502-4 (PMC12913794; doi:10.1038/s41413-025-00502-4)
Supplement: Supplementary file 20 — Supplementary information [file 41413_2025_502_MOESM20_ESM.docx]

**Supplementary information**

**Fig.S1. Inflammatory macrophages accumulate in the synovium of the OA model.**

A, A schematic diagram illustrating the experimental design. B, Representative images of HE staining in the synovium of SD rats at 8 weeks and 16 weeks after sham or OA surgery (up), scale bar: 200 μm. Higher magnification images show dramatic synovitis changes (down), scale bar: 20 μm. C, Quantitative data of synovitis score in the synovium with HE staining. n=8 for each group. D,E, Representative images of F4/80 with CD206 (D) or iNOS(E) in the synovium of SD rats at 8 weeks and 16 weeks after sham or OA surgery (up), scale bar: 100 μm. F,G, The quantification of F4/80 with CD206 (F) or iNOS (G) in the synovium with IF staining. n=8 for each group. H,I, Quantitative analysis of COL2A1 (H) and MMP13 (I) were assessed by IF staining in C28/I2 cells treated with conditioned medium from THP-1 cells. n=4 for each group. Scale bar: 20 μm. DAPI: 4,6-diamidino-2-phenylindole. r.f.u., relative fluorescence units. J,K, Representative images and quantitative analysis of COL2A1 (J) and MMP13 (K) were assessed by IF staining in C28/I2 cells treated with EVs from THP-1 cells. n=4 for each group. Scale bar: 20 μm. DAPI: 4,6-diamidino-2-phenylindole. r.f.u., relative fluorescence units. One-way ANOVA &Tukey HSD post hoc test (normal distribution) and Kruskal-Wallis Test & Dunn’s test (non-normal distribution) were used for multiple comparisons. * *p*<0.05, ** *p*<0.01, *** *p*<0.001, **** *p*<0.001, ns not significant.

**Fig.S2. Synovial pro-inflammatory macrophages-derived EVs downregulate the autophagy function of** **chondrocytes in OA progression.**

A, Identification of M1 polarization of THP-1 cells by flow cytometry. B, Representative transmission electronic microscope (TEM) images of EVs derived from M1-polarized THP-1 cells. Scale bar: 100 nm. C, Nanoparticle tracking analysis (NTA) for measuring the size distribution and concentration of EVs derived from M1-polarized THP-1 cells. D, Gating strategy for blood monocyte of wt rats. Blood monocytes were defined as CD45^+^, CD11b^+^. E, Identification of M1 polarization of BMDMs in rat models by flow cytometry. F, Nanoparticle tracking analysis (NTA) for measuring the size distribution and concentration of EVs derived from M1-polarized BMDMs in rat models. G, GSEA enrichment analysis of control group and EVs^M1^ group.

**Fig.S3. Synovial pro-inflammatory macrophages-derived EVs downregulate the autophagy function of chondrocytes in OA progression.**

A, RNA-Seq analysis was conducted in total articular cartilage mRNA of OA rats, which was extracted from the control group (PBS-treated, n = 3) and EVs^M1^ group (EVs^M1^-treated, n = 3). Volcanic map of different expression genes (DEGs) between the control group and EVs^M1^ group. Red spots represent up-regulated genes, and blue spots represent down-regulated genes. B, KEGG enrichment of articular cartilage in OA rats from the control group or EVs^M1^ group. C, Transmission electron microscopy (TEM) image of autophagic vesicles in articular cartilage of OA rats. The red arrow indicates the cell bilayer membrane structure of autophagic vesicles. Scale bar: 1 μm. D, Quantification of autophagic vesicles in articular cartilage of OA rats with TEM detection. n=7 for each group. E-F, Representative images (E) and quantitative analysis (F) of LC3 dots per cell in C28/I2 cells treated with conditioned medium from THP-1 cells. n=4 for each group. Scale bar: 10 μm. G,H, Representative images (G) and quantitative analysis (H) of LC3 dots per cell in C28/I2 cells treated with EVs from THP-1 cells. n=4 for each group. Scale bar: 10 μm. One-way ANOVA &Tukey HSD post hoc test (normal distribution) and Kruskal-Wallis Test & Dunn’s test (non-normal distribution) were used for multiple comparisons. * *p*<0.05, ** *p*<0.01, *** *p*<0.001, **** *p*<0.001, ns not significant.

**Fig.S4. Synovial pro-inflammatory macrophages-derived EVs miR-155-5p regulates the autophagy function of chondrocytes.**

A, The hub DEGs in GSE175961 (miRNAs of the cartilage between KOA patients and healthy controls), GSE33453 (miRNAs of M0/M1 BMDMs in mice) and the our results (miRNAs of EVs^M1-BMDMs^ in OA rats). B, Enrichments of miR-155-5p in EVs derived from M0 and M1-polarized BMDMs, rat chondrocytes and M0 and M1-polarized BMDMs determined by qRT-PCR. C, Enrichments of miR-155-5p in articular cartilage and synovium of SD rats at 8 weeks and 16 weeks after sham or OA surgery. D, E, Quantification of expression of COL2A1 (D) and MMP13 (E) in articular cartilage of OA rats with IF staining. n=7 for each group. F, Representative image of DiI-labelled EVs derived from M1-polarized BMDMs absorbed by rat chondrocytes. Scale bar: 10 μm. G, qRT-PCR analysis of of miR-155-5p in articular cartilage and synovium from individuals with OA who underwent total hip joint replacement surgeries (*n* = 8) and healthy individuals (*n* = 4). H, Western blot showing autophagy-related proteins, including Beclin1, LC3A/B, ATG3, ATG7, and LAMP1 in the articular cartilage of sham or OA rats treated with PBS, EVs^M1^, antagomiR-155-5p, and antagomiR-NC. One-way ANOVA &Tukey HSD post hoc test (normal distribution) and Kruskal-Wallis Test & Dunn’s test (non-normal distribution) were used for multiple comparisons. Two-tailed Student's t test (normal distribution) and Mann-Whitney U test (non-normal distribution) were used for comparisons between the two groups.

**Fig.S5. Synovial pro-inflammatory macrophages-derived EVs miR-155-5p targets GSK‑3β/ mTORC1 axis in OA chondrocytes.**

A, Representative image of FAM-labelled miR-155-5p absorbed by C28/I2 cells. Scale bar: 10 μm. B, The relative levels of miR-155-5p in C28/I2 cells transfected with miR-155-5p mimic and miR-NC were assessed using qRT-PCR. C, Western blot showing the mTORC1 signaling pathway-related proteins, including: GSK‑3β, p70 S6, P-p70 S6, S6, and P-S6 in C28/I2 cells transfected with miR-155-5p mimic, miR-NC, inhibitor and inhibitor NC. D, Western blot showing the protein levels of GSK‑3β in GSK‑3β^KD^/SCR^KD^ C28/I2 cells. One-way ANOVA &Tukey HSD post hoc test (normal distribution) and Kruskal-Wallis Test & Dunn’s test (non-normal distribution) were used for multiple comparisons. * *p*<0.05, ** *p*<0.01, *** *p*<0.001, **** *p*<0.001, ns not significant.

**Fig.S6. Western blot showing the protein levels of FMRP in FMRP^OE^/Vector^OE^ and FMRP^KD^/SCR^KD^ THP-1 cells.**

**Fig.S7. Genetic knockout of miR-155-5p in synovial macrophages retards OA progression.**

A, PCR genotyping using tail DNA. miR-155^flox^ KO, 200bp; miR-155^flox^ WT, 132bp; LysM^Cre^, 721bp. B, Gating strategy for blood monocyte of miR-155 CKO mice. Blood monocytes were defined as CD45^+^, CD11b^+^, Gr1^+^, F4/80^+^. C, Enrichments of miR-155-5p in inflammatory macrophages derived from BMDMs in miR-155 CKO mice and Control mice. n=6 for each group. D, The percentage of BMDMs-derived M1-like macrophages (CD45^+^, CD11b^+^, F4/80^+^, CD86^+^) from *LysM^Cre^*; *miR-155 ^fl/fl^* (CKO) and *miR-155 ^fl/fl^* (Control) mice was determined. E, Enrichments of miR-155-5p in EVs derived from M0 and M1-polarized macrophages of THP-1 cells, C28/I2 cells and M0/M1-polarized macrophage of THP-1 cells determined by qRT-PCR. n=6 for each group. F, Enrichments of miR-155-5p in EVs derived from M0 and M1-polarized macrophages in miR-155-5p knockdown (KD) THP-1 cell lines and miR-NC knockdown (KD) THP-1 cell lines (Control), determined by qRT-PCR. n=4 for each group. G, Breeding strategy of miR-155 CKO (*Col2a1^Cre^*; *miR-155^fl/fl^*) mice. H, SO & FG staining for keen joints in Control and *Col2a1^Cre^*; *miR-155^fl/fl^* mice with DMM surgery, scale bar: 200 μm. I, Quantification of OARSI score was performed using histological sections. n=7 for each group. J, Western blot showing the protein levels of COL2A1, SOX9, MMP13 in primary mice chondrocytes. K, A schematic diagram illustrating the experimental design. L, SO & FG staining for keen joints in *LysM^Cre^*; *miR-155 ^fl/fl^* mice with DMM surgery (up), scale bar: 200 μm. Higher magnification images show dramatic articular cartilage changes (down), scale bar: 50 μm. M, Quantification of OARSI score was performed using histological sections. n=6 for each group. N, IHC staining of knee joint sections showing expression of COL2A1 in articular cartilage (up), scale bar: 200 μm. Higher magnification images show dramatic articular cartilage changes (down), scale bar: 50 μm. O, Quantification of expression of COL2A1 in articular cartilage of OA mice with IHC staining. n=6 for each group. Two-tailed Student's t test (normal distribution) and Mann-Whitney U test (non-normal distribution) were used for comparisons between the two groups. One-way ANOVA &Tukey HSD post hoc test (normal distribution) and Kruskal-Wallis Test & Dunn’s test (non-normal distribution) comparisons. * *p*<0.05, ** *p*<0.01, *** *p*<0.001, **** *p*<0.001, ns not significant.

**Fig.S8. Genetic knockout of miR-155-5p in synovial macrophages retards OA progression.**

A,B,C, Representative images (A) and quantitative analysis of COL2A1 (B) and MMP13 (C) were assessed by IF staining in C28/I2 Cells treated with EVs derived from M0 and M1-polarized macrophages in miR-155-5p/miR-NC knockdown (KD) THP-1 cell lines and normal THP-1 cell (Control). n=4 for each group. Scale bar: 20 μm. DAPI: 4,6-diamidino-2-phenylindole. r.f.u.: relative fluorescence units. D, Quantitative analysis of LC3 dots per cell in C28/I2 Cells treated with EVs derived from M0 and M1-polarized macrophages in miR-155-5p/miR-NC knockdown (KD) THP-1 cell lines and normal THP-1 cell (Control). n=4 for each group. One-way ANOVA &Tukey HSD post hoc test (normal distribution) and Kruskal-Wallis Test & Dunn’s test (non-normal distribution) were used for multiple comparisons. * *p*<0.05, ** *p*<0.01, *** *p*<0.001, **** *p*<0.001, ns not significant.

**Fig.S9. Surface modification of ADSCs-derived EVs.**

A, Schematic diagram of the plasmid constructs containing Lamp2b and MAP-Lamp2b, and the engineering EVs derived from ADSCs. B, Nanoparticle tracking analysis (NTA) for measuring the size distribution and concentration of MAP-EVs^ADSCs^. C, Transmission electron microscopy (TEM) analysis for the morphology of MAP-EVs^ADSCs^ (Scale bar: 200 nm). D, Western blot analysis of protein markers CD9, CD81, Alix, Tsg101, and Calnexin in whole cell lysates (WCLs) and purified EVs including EVs^ADSCs^ and MAP-EVs^ADSCs^. E,F, Enrichments of miR-155-5p in the synovium (E) and articular cartilage (F) of the aforementioned rats treated with engineering EVs. n=6 for each group. One-way ANOVA &Tukey HSD post hoc test (normal distribution) and Kruskal-Wallis Test & Dunn’s test (non-normal distribution) were used for multiple comparisons. * *p*<0.05, ** *p*<0.01, *** *p*<0.001, **** *p*<0.001, ns not significant.
